# Genome sequence of the pattern forming *Paenibacillus vortex *bacterium reveals potential for thriving in complex environments

**DOI:** 10.1186/1471-2164-11-710

**Published:** 2010-12-17

**Authors:** Alexandra Sirota-Madi, Tsviya Olender, Yael Helman, Colin Ingham, Ina Brainis, Dalit Roth, Efrat Hagi, Leonid Brodsky, Dena Leshkowitz, Vladimir Galatenko, Vladimir Nikolaev, Raja C Mugasimangalam, Sharron Bransburg-Zabary, David L Gutnick, Doron Lancet, Eshel Ben-Jacob

**Affiliations:** 1 The Sackler School of Physics and Astronomy, Tel Aviv University, P.O. Box 39040, Tel Aviv 69978, Israel; 2 The Sackler School of Medicine, Tel Aviv University, P.O. Box 39040, Tel Aviv 69978, Israel; 3 Department of Molecular Genetics, Weizmann Institute of Science, Rehovot 76100, Israel; 4 Department of Plant Pathology and Microbiology, The Robert H. Smith Faculty of Agriculture, Food and Environment, The Hebrew University of Jerusalem, Rehovot 76100, Israel; 5 Laboratory of Microbiology, Wageningen University, Wageningen 6703 HB, Netherlands; 6 Laboratory of System Biology and Analysis of High-throughput data, Institute of Evolution, University of Haifa, Haifa 31905, Israel; 7 Department of Biological Services, Weizmann Institute of Science, Rehovot 76100, Israel; 8 Department of Mathematical Analysis, Faculty of Mechanics and Mathematics, Moscow State University, Moscow 119991, Russia; 9 A.N. Belozersky Institute of Physico-Chemical Biology, Moscow State University, Moscow 119991, Russia; 10 Genotypic Technology (P) Ltd, Bangalore 560094, India; 11 Department of Molecular Microbiology and Biotechnology, Tel Aviv University, Tel Aviv 69978, Israel; 12 The Center for Theoretical and Biological Physics, University of California San Diego, La Jolla, California 92093, USA

## Abstract

**Background:**

The pattern-forming bacterium *Paenibacillus vortex *is notable for its advanced social behavior, which is reflected in development of colonies with highly intricate architectures. Prior to this study, only two other *Paenibacillus *species (*Paenibacillus *sp. JDR-2 and *Paenibacillus larvae*) have been sequenced. However, no genomic data is available on the *Paenibacillus *species with pattern-forming and complex social motility. Here we report the *de novo *genome sequence of this Gram-positive, soil-dwelling, sporulating bacterium.

**Results:**

The complete *P. vortex *genome was sequenced by a hybrid approach using 454 Life Sciences and Illumina, achieving a total of 289× coverage, with 99.8% sequence identity between the two methods. The sequencing results were validated using a custom designed Agilent microarray expression chip which represented the coding and the non-coding regions. Analysis of the *P. vortex *genome revealed 6,437 open reading frames (ORFs) and 73 non-coding RNA genes. Comparative genomic analysis with 500 complete bacterial genomes revealed exceptionally high number of two-component system (TCS) genes, transcription factors (TFs), transport and defense related genes. Additionally, we have identified genes involved in the production of antimicrobial compounds and extracellular degrading enzymes.

**Conclusions:**

These findings suggest that *P. vortex *has advanced faculties to perceive and react to a wide range of signaling molecules and environmental conditions, which could be associated with its ability to reconfigure and replicate complex colony architectures. Additionally, *P. vortex *is likely to serve as a rich source of genes important for agricultural, medical and industrial applications and it has the potential to advance the study of social microbiology within Gram-positive bacteria.

## Background

**Figure 1:**
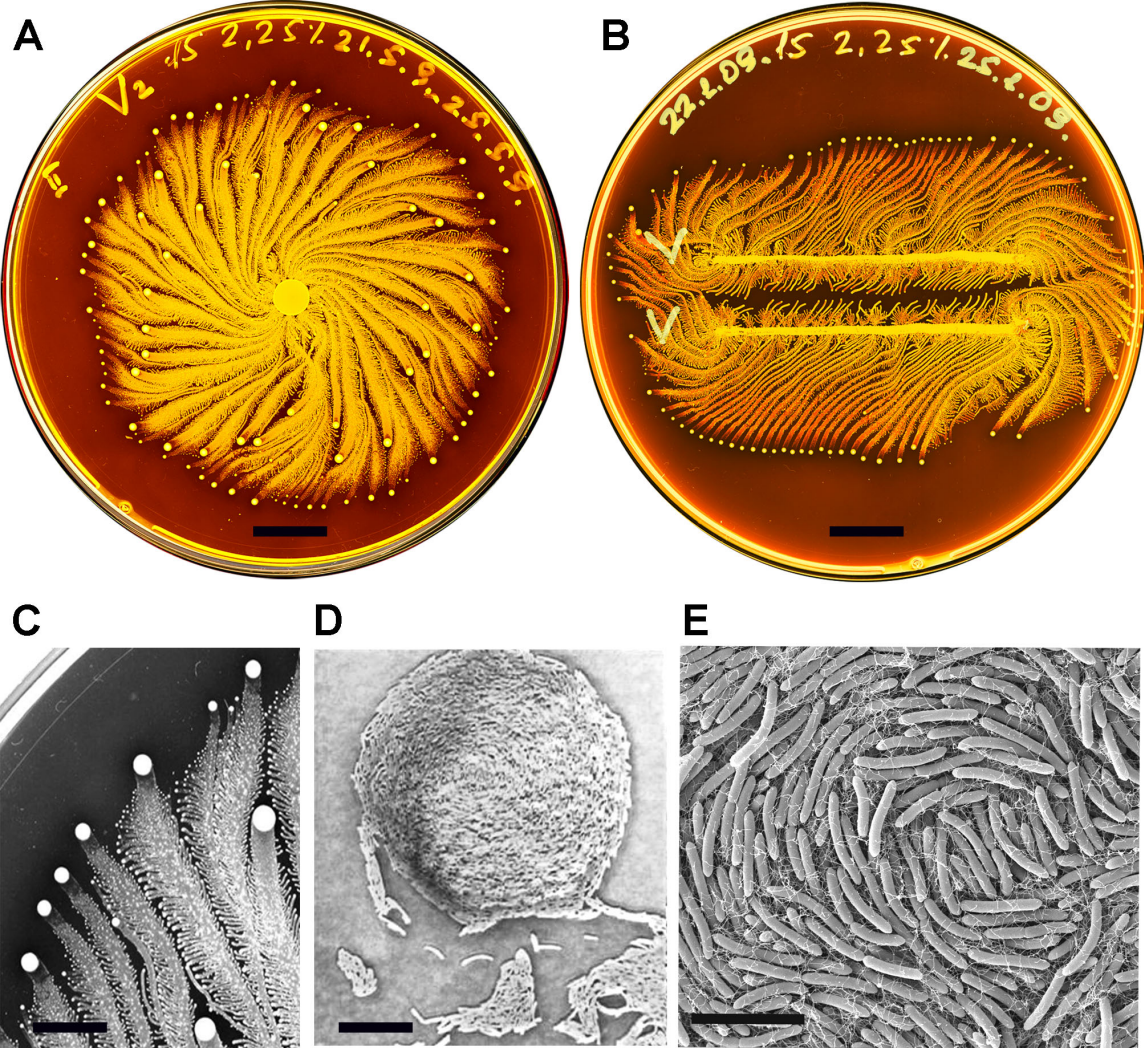
**Colony organization of the *P. vortex *bacteria**. (A) Whole colony view of *P. vortex*, when grown on 15 g/l peptone and 2.25% (w/v) agar for four days. The bright yellow dots are the vortices as described in the text. (B) Two colonies of *P. vortex*, inoculated in two parallel lines, on 15 g/l peptone and 2.25% (w/v) agar. Structure flexibility of the colony architecture is illustrated. The colonies in A and B were grown in a Petri dish size 8.8 cm and stained with Coomassie dyes (Brilliant Blue). The colors were inverted to emphasize higher densities using the brighter shades of yellow. (C) Magnification of x20 into the colony pattern and vortex progress. (D) An example of a mature individual vortex magnification x500. (E) Scanning electron microscope (SEM) observation of *P. vortex *illustrating a typical bacteria arrangement in the center of a vortex. Notable, that each individual bacterium has a curvature. Scale bar in (A-B) is 1 cm, in (C) is 500 μm, in (D) is 20 μm and in (E) is 5 μm.

*Paenibacillus vortex *strain V453 [1] is a bacterial species discovered in the early 90's [2]. It is a social microorganism that forms colonies with remarkably complex and dynamic architectures (Figure 1) [2-4]. The genus *Paenibacillus*, including *P. vortex*, was originally considered a part of the genus *Bacillus *but was later reclassified as a separate genus in 1993 [5]. These facultative anaerobic, spore-forming bacteria are found in a variety of heterogeneous environments, such as soil, rhizosphere, insect larvae, and clinical samples [6-9].

To face the challenges posed by these environments, *Paenibacillus *spp. produce a wealth of enzymes and proteases as well as a great variety of antimicrobial substances that affect a wide range of microorganisms [10-12]. The possession of these advanced defensive and offensive strategies render the *Paenibacillus *spp. bacteria as a rich source of useful genes for agricultural, medical, industrial applications. Despite this potential, genome sequencing of *Paenibacillus *spp. to date is limited and is currently available only for two species *Paenibacillus larvae *and *Paenibacillus *sp. JDR-2.

A successful behavioral strategy utilized by some *Paenibacillus spp*. is to cooperatively form and develop large and intricately organized colonies of 10^9^-10^12 ^cells. Being part of a large cooperative, the bacteria can better compete for food resources and be protected against antibacterial assaults [3,13]. Two of the most fascinating pattern-forming *Paenibacillus spp*. bacteria, are *P. vortex *[3,14] and *P. dendritiformis *[3,15]. Under laboratory growth conditions, these bacteria can develop, like other social bacteria, colonies that behave much like a multi-cellular organism, with cell differentiation and task distribution [16-19] (see also Additional file 1 section I).

*P. vortex *possesses advanced social motility employing cell-cell attractive and repulsive chemotactic signaling and physical links (Additional file 1 section I). When grown on soft surfaces, the collective motility is reflected by the formation of foraging swarms [14] that act as arms sent out in search for food (Additional file 1 section I and Additional files 2, 3, 4, 5, 6). These swarms have an aversion to crossing each other's trail and collectively change direction when food is sensed. The swarms can even split and reunite when detecting scattered patches of nutrients [14].

When grown on hard surfaces, *P. vortex *generates special aggregates of dense bacteria that are pushed forward by repulsive chemotactic signals sent from the cells at the back (see Additional file 1 section I). These rotating aggregates (termed vortices), are similar to the rotating bacteria groups generated by *Paenibacillus alvei *[20] and *Bacillus circulans *[21], pave the way for the colony to expand. The vortices serve as building blocks of colonies with special modular organization (Figure 1 and Additional file 1 section I).

Accomplishing such intricate cooperative ventures requires sophisticated cell-cell communication [3,19,22-24]. Communicating with each other, bacteria exchange information regarding population size, a myriad of individual environmental measurements at different locations, their internal states and their phenotypic and epigenetic adjustments [25]. The bacteria collectively sense the environment and execute distributed information processing to glean and assess relevant information [3,19,25]. Next, the bacteria respond accordingly, by reshaping the colony while redistributing tasks and cell differentiations, and turning on defense and offense mechanisms [3,16-19,25,26], thus achieving better adaptability to heterogeneous environments [3]. Such collective, decentralized, adaptive decision making is a form of swarm intelligence, a term originally derived from cybernetics but applicable to some aspects of colonial organisms including ants, birds, humans and bacteria [27-29]. In terms of collective social behaviour, *P. vortex *has been studied extensively at the level of mathematical modeling [3,30-32] and now requires a sequenced genome to connect this approach with the underlying genetics.

Comparative genomic analysis revealed that bacteria successful in heterogeneous and competitive environments often contain extensive signal transduction and regulatory networks [33-35]. It is likely that advanced social behavior [19] and elevated collective adaptability [3] are underpinned by a highly developed signal transduction system consisting of modular domains forming a network of sensors, transducers and responders [34,36,37].

In this report we present the *de novo *genome sequence of the *P. vortex*, which was obtained by utilizing a hybrid deep-sequencing approach using 454 and Illumina techniques [38,39]. We further performed detailed comparative genomic analysis with a dataset of 500 complete bacterial genomes to discover *P. vortex *unique properties. The results revealed that *P. vortex *has one of the highest number of signal transduction genes among all the Gram-positive bacteria in the dataset. Only two other Gram-positive bacteria strains, the *Paenibacillus *sp. JDR-2 and the *Geobacillus *sp. Y412MC10, have more TCS genes. These two species and *P. vortex *have equal normalized combined score of TCS, TFs, transport and defense related genes (see material and methods), which is significantly higher than the combined score of all other bacteria in the data set.

The analysis also unveiled genes required for competition over resources (e.g. iron, amino acids and sugars), for producing offensive compounds (antibiotics and lytic enzymes) and for defense (resistance to antibiotics and other toxins). These genes can support traits needed for thriving in the heterogeneous and highly competitive environments.

## Results

### Sequencing of the P. vortex genome

#### Hybrid assembly

*De novo *assembly of the *P. vortex *genome was obtained using the two leading deep-sequencing technologies: Roche 454 Genome Sequencer (GS 20) [40] and Illumina Genome Analyzer (GA) [41]. Using the Roche 454 and the Illumina GA technologies, 19× coverage of single reads and 270× total average coverage of single and paired-end mapped reads was produced respectively (Table 1). The reads from each technology were first assembled separately and then joined into a hybrid assembly to improve scaffold size and quality (Additional file 1 section II). The hybrid assembly (Additional file 1 Figure S6) contains 56 scaffolds totaled 6,385,925 bp with N50 scaffold size of 213,399 bp and largest scaffold of 699,613 bp. Notably, the contigs from the two technologies could be joined easily as no miss-assemblies were detected between the two sets of contigs. The first version of the Whole Genome Shotgun project described in this paper has been deposited at [GenBank: ADHJ00000000].

**Table 1:** Summary of the sequencing results obtained from each of the technologies.

	Roche 454	Illumina GA
Average read length (nt)	100	36
Total no. single reads	1,192,566	30,779,535
Total no. paired-end reads	-	22,264,798
Average depth-coverage of mapped reads	19×	270×
Total contigs (> 500 nt)	227	224
Number of scaffolds (> 500 nt) using 454 and Illumina	56	

#### Assembly accuracy and completeness

To estimate the accuracy and the completeness of the hybrid assembly we performed detailed comparison between the 454 and the Illumina contigs. The results show that the 454 contigs covered 99.93% of the hybrid assembly with an average distance between contigs comprising the hybrid scaffolds of -5 bp and total 890 bp missing from the hybrid assembly (Additional file 1 Figure S6 B, C, D). The Illumina contigs covered 99.81% of the hybrid assembly with average distance between contigs of -10 bp and missing total 4,500 bp (Additional file 1 Table S3). The overall sequence identity between the two technologies was 99.8%. These results and the fact that there were no miss-assemblies demonstrate that although the *P. vortex *assembly is in several contigs, it provides complete genome coverage and with an extremely high accuracy (Additional file 1 section II).

#### Scaffolds ordering

**Figure 2:**
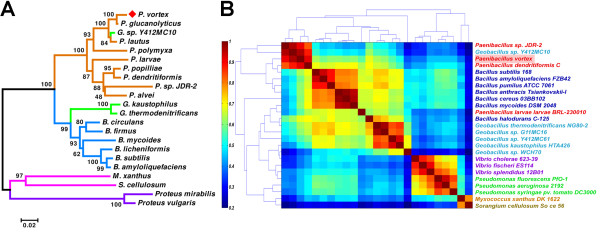
***P. vortex *classification based on phylogenetic analysis and function COG clustering**. (A) Phylogenetic tree based on 16 S rRNA. The abbreviated genera *P *- *Paenibacillus*, *B *- *Bacillus*, *G *- *Geobacillus*, *M *- *Myxococcus*, *S *- *Sorangium*. Bootstrap values are shown next to the branches. (B) COG profile analysis of 25 bacterial species using Pearson correlation matrix. Abundance profile vector of 4,873 COGs was calculated for 25 different bacterial species representing various taxons. The computed Pearson correlation matrix was ordered using the dendrogram clustering algorithm to identify clusters and was color-coded from dark blue representing very low correlation to dark red representing very high correlation. The *Geobacillus sp*. Y412MC10 is clustered with *Paenibacillus *species (upper left red square) and not with the rest of the *Geobacillus *species. Additionally, *Paenibacillus larvae *is not part of the *Paenibacillus *cluster.

To obtain a putative order of the *P. vortex *scaffolds, we used *Geobacillus*. sp. Y412MC10 genome [Refseq: NC_013406] as a reference and ordered the *P. vortex *scaffolds accordingly. Our preliminary genomic comparison identified *Geobacillus *sp. Y412MC10 as the closest bacteria with a complete genome to *P. vortex*. The identification was based on phylogenetic analysis of 16 S rDNA placing the *Geobacillus *sp. Y412MC10 within the *P. vortex *clade (Figure 2A) and further supported by genomic clustering of Cluster of Orthologous Groups (COG) profiles [42,43] (Figure 2B). BLASTn comparison results of *P. vortex *genome vs. *Geobacillus sp*. Y412MC10, revealed that 2/3 of the *P. vortex *genome could be matched to *Geobacillus sp*. Y412MC10 with an average sequence identity of 86.69% over a mean alignment length of 783 bp (Additional file 1 Figure S7).

#### General genome statistics

**Figure 3:**
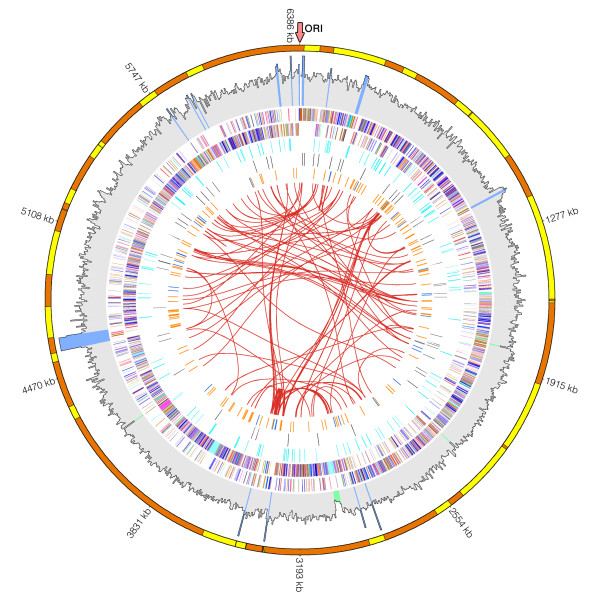
**Genome atlas of *P. vortex***. Circles indicated from outside to inside: (1) *P. vortex *56 contigs ordered by *Geobacillus sp*. Y412MC10. The contigs marked in yellow and orange alternately. (2) Illumina sequencing base coverage histogram. The average value was calculated for sliding window of 5000 bp. Areas with extremely high and low coverage: mean ± 2 stdev (270 ± 40) were marked in blue and green respectively. The rest was marked grey. (3) *P. vortex *COG categories on the forward strand (+). (4) *P. vortex *COG categories on the reverse strand (-). (5) 210 two-component system genes are shown in orange. (6) 73 predicted ncRNAs genes are indicated in black. (7) Local inverted repeats marked blue and tandem repeats marked orange. (8) Global repeats identified in two or more positions are connected using red lines.

The *P. vortex *genome is composed of a circular chromosome (6,385,925 bp) with an average G+C content of 48.7% (Figure 3). A total of 6,437 open reading frames (ORFs) were identified covering 86% of the *P. vortex *genome (Table 2). Among the predicted ORFs, 4,475 (70%) were assigned with a putative function, whereas 1,962 (30%) were identified as hypothetical proteins. We identified 73 non-coding RNA genes and 54 tRNA genes predicted to incorporate 18 amino acids into polypeptides. The tentative location of the origin of replication (ORI) was identified based on its proximity to *dnaA *gene, known to serve as a transcription initiator protein [44].

**Table 2:** Genomic features of the P. vortex genome.

Genome size (nt)	6,385,925
G+C content (%)	48.7
Protein-coding sequences	6,437
Genes with assigned function	4,475
Genes with unknown function	1,962
Average CDS size (nt)	852
Percent of coding region	86
rRNA	3
ncRNA	73
tRNA	54

#### Repetitive sequences

We have identified several types of repetitive sequences: 184 global repeats (sequence that is present in at least two copies in two different locations), 32 local inverted repeats and 231 tandem repeats within the *P. vortex *genome (Figure 3) (for methods see Additional file 1 section VII). Such sequences were suggested to play an important functional role in genome plasticity [45], by means of homologous recombination (HR), horizontal transfer or transposition in the genome [46-48]. HR has relevant roles in DNA repair, chromosome segregation and generation of genetic variation. Crossover events might produce genome rearrangements, such as deletions, leading to the loss of all genetic information in that region or duplications which could increase the amount of genetic information [49]. Additionally, repeats located within regulatory regions might constitute an on/off switch of gene expression at the transcriptional level [50]. Similarly, repeats located within coding regions can induce a premature ending of translation when a mutation changes the number of repeats [51]. However, detailed mechanisms and functions of most repeats are still unknown.

Repetitive sequences are the major reason for the difficulty we encountered in finishing the genome assembly into a complete sequence. Analysis of the scaffold ends (100 bp of each end) revealed that 78% of them have repetitive sequences that are on average 37 bp long and could be mapped on average onto 5 different scaffold ends.

We note that some regions in the *P. vortex *genome have an extremely high coverage (see areas marked in blue, second circle, Figure 3). Although, the assembly algorithms tend to collapse the highly identical repetitive sequences into one copy, high coverage in that specific area might serve as a signature for identifying regions present in several copy numbers in the genome [52]. For example, the ribosomal unit (16 S, 23 S and 5S) has approximately 5 times higher coverage than the average, suggesting that this unit appears approximately 5 times in the *P. vortex *genome. Interestingly, the *Geobacillus *sp. Y412MC10 has 8 copies of the ribosomal unit.

#### Functional validation by custom microarray

We used specially designed Agilent custom microarray submitted to EMBL-EBI [ArrayExpress: E-MEXP-3019] to validate the annotation. The microarray (Additional file 1 section IV) includes 105,000 oligos of 60 bp long, which corresponds to all the predicted ORFs and the intergenic regions.

Hybridization of the genomic DNA validated 91,324 probes (88%) of the total designed probes and no missed regions were found (see Additional file 1 section IV for more details). Hybridization of the pooled RNA from different growth conditions confirmed 4,701 (73%) of the predicted ORFs. The remaining 1,736 (27%) ORFs were not detectable under the tested conditions. Out of those, 1,064 ORFs have an assigned putative function and 672 are hypothetical. Hybridization of predicted 73 non-coding RNAs located within the intergenic regions, confirmed 43 (58%).

### Comparative Analysis

We performed detailed comparative analysis between the *P. vortex *genome and a set of 500 complete bacterial genomes of 2-10 Mbp (Additional file 7). Bacterial genomes available with draft sequence were not included in the analysis. Specifically, we focused on a reduced set of 261 genomes with genome size of 4-8 Mbp (closer to the *P. vortex *genome size) and a subset of 50 soil bacteria genomes within this group (Additional file 8). The comparison was done with regard to four gene systems which are related to complex bacterial lifestyle and adaptability to fluctuating environments: two-component systems, transcription factors, defense mechanisms and transport systems.

#### Two-component system (TCS)

**Figure 4:**
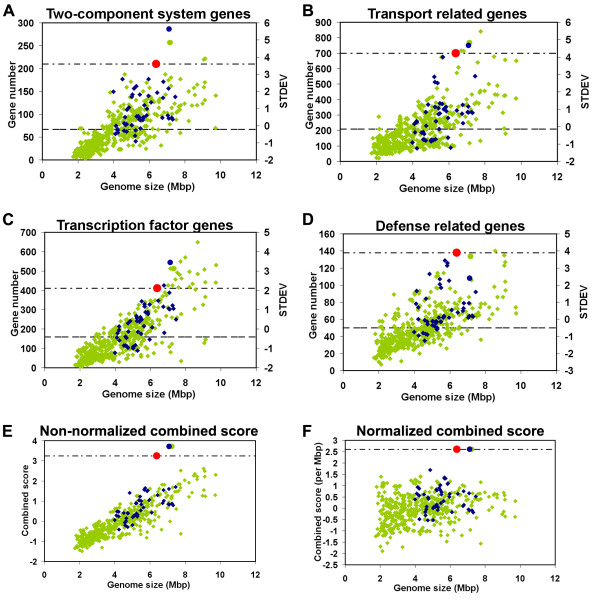
**Statistics for 500 bacterial genomes as a function of genome size is presented**. Gene number for 50 soil bacterial genomes sized between 4-8 Mbp marked in blue and the rest of the genomes marked in green. *Paenibacillus *sp. JDR-2 is marked in blue circle; *Geobacillus *sp. Y412MC10 is marked in green circle; *P. vortex *is marked in red circle and its value as dotted line. STDEV for A-C graphs is presented on the right side of the axis and the mean value is presented in the dashed line. (A) Two-component system (TCS) genes plot is presented. *P. vortex *possesses 210 TCS genes. (B) Transport related genes are presented. *P. vortex *possesses 700 such genes. (C) Plot of transcription factor genes. *P. vortex *possesses 411 such genes. (D) Plot of genes related to defense mechanisms. *P. vortex *possesses 138 such genes. (E) Non-normalized combined score as function of genome size. The combined score is calculated as an average of the stdev of TCS, TF, transport and defense genes for the dataset of 500 bacterial genomes. (F) Combined score normalized to genome size.

Using Pfam motifs [53] we identified a total of 210 TCS related genes in the *P. vortex *genome; 103 response regulators (RRs), 97 histidine-kinases (HKs) and 10 hybrid kinases. The number of TCS genes was linear with genome size in agreement with [35]. Among the 500 bacterial genomes, *P. vortex *was at the upper 1% of the population (Figure 4A, Additional file 1 Figure S12), along with two Gram-positive bacteria strains *Paenibacillus sp*. JDR-2 (7.08 Mbp) and *Geobacillus sp*. Y412MC10 (7.12 Mbp) and two Gram-negative bacteria strains, the predator myxobacterium *M. xanthus *(9.13 Mbp) and the cyanobacterium *N. punctiforme *PCC 73102 (9.05 Mbp). Our results show that similarly to the absolute gene numbers, the relative gene numbers of the tested categories in *P. vortex *genome is also significantly higher compared to the rest of the 500 genomes (Additional file 1 Figure S11).

Structural classification of the *P. vortex *RRs according to previously proposed scheme [54] revealed relatively high number of 37 OmpR family and 30 AraC family DNA-binding response regulators. Class organization of the *P. vortex *TCS proteins as described in [35] revealed 150 HK-RR paired, 32 orphaned (isolated) and 21 in complex gene clusters (for more details see Additional file 1 section VII). Neighborhood analysis of the TCS surrounding genes revealed that 101 (30%) are transport related genes, 46 (12.6%) have regulatory functions (mainly consist of transcription factors), and 35 (9.6%) belong to the energy metabolism category (mainly employing biosynthesis and degradation of polysaccharides).

#### Transcription Factors (TFs)

Using the method described in [55], we identified a total of 411 TFs in *P. vortex *genome, which placed it at the upper 5% of the 500 bacteria set (Figure 4C). This number is considerably higher than the average 158 ± 111 TFs among the 500 bacterial genomes and higher than the average 208 ± 92 TFs among the subset of 261 genomes with size 4-8 Mbp sizes. Among the subset of 50 soil bacteria genomes, only two strains, *Paenibacillus sp*. JDR-2 (7.08 Mbp) and *Delftia acidovorans SPH-1 *(6.76 Mbp) have a higher number of TFs genes. We note that an overall linear dependence between the TFs and the genome size was found (Figure 4C).

#### Transporter Genes

*P. vortex *encodes an extensive set of 700 transport related genes. Among the 500 bacterial genomes, *P. vortex *was at the upper 1% of the population (Figure 4B), along with additional five strains *Rhizobium leguminobarum bv. viciae *(7.75 Mbp), *Geobacillus sp*. Y412MC10 (7.12 Mbp), *Paenibacillus sp*. JDR-2 (7.08 Mbp), *Sinorhizobium meliloti 1021 *(6.7 Mbp) and *Sinorhizobium medicae WSM419 *(6.8 Mbp). About a third, 258 (35%) of the genes are involved in carbohydrate transport, 42 genes encode components of iron transporters, 23 genes encode components of amino acid transporters and 39 genes encode components of oligo/dipeptide transporters. The latter could be used as nutrient sources, as well as signal molecules regulating bacterial development, virulence, and conjugal plasmid transfer [56].

#### Defense Mechanisms

The *P. vortex *genome contains 138 genes related to resistance against inhibitory substances such as antibiotics, copper, aluminium, arsenic and toxic anions (Figure 4D). The proximity of TCS genes to ABC transporters is known to form specific and efficient detoxification units [37]. Out of the 138 genes, 90 are transporter-encoding genes. Non-transport related genes include antibiotic resistance encoding genes such as penicillin binding proteins, beta-lactames, chloramphenicol posphotransferases/acetyltransferases, *vanZ *and *vanW *glycopeptide antibiotics resistance genes. Apart from *Streptomyces griseus NBRC 13350 *(8.54 Mbp), *P. vortex *harbors the highest number of defense related genes among the 500 analyzed genomes. Additionally, *P. vortex *has the highest number of these genes compared to the subset of 261 genomes with a 4-8 Mbp genome size (the average for this subset is 60 ± 20).

#### The combined score

When compiling the four indices into a combined score, *P. vortex *and two other Gram-positive bacteria strains, the *Paenibacillus *sp. JDR-2 and the *Geobacillus *sp. Y412MC10 stand out among the 500 genomes in the dataset (Figure 4E). These two species and *P. vortex *have equal normalized combined score (Figure 4F), which is significantly higher than the combined score of all other bacteria in the dataset.

### Motility and Chemotaxis

**Figure 5:**
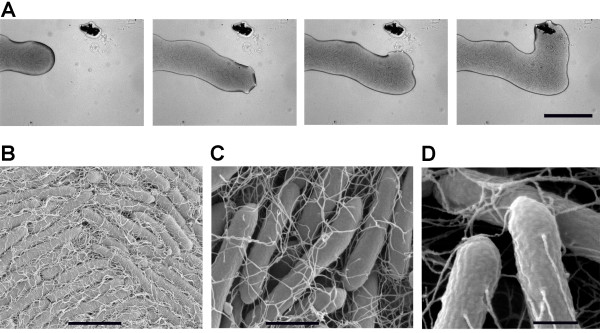
**Flagella mediated physical interactions between *P. vortex *bacteria**. (A) Snapshots from a video clip of a branch of a *P. vortex *colony moving on Mueller-Hinton agar (0.3% w/v) (x50 magnification, scale bar 200 μm) towards a target of extracellular material (dark spot). See Movies S4-S5 and [14] for more details. (B-D) Scanning electron microscope (SEM) pictures of bacteria close to the center of the vortex. Scale bar in (B) is 4 μm and in (C) is 1 μm. The bacteria in (D) are around 400 nm wide and the flagella are 20 to 35 nm wide.

**Figure 6:**
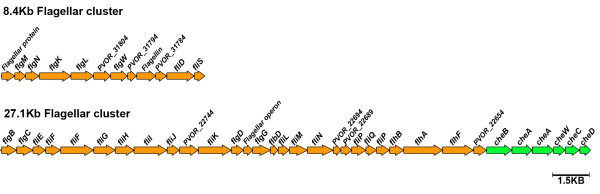
**Partial map of the *P. vortex *two biggest flagellar and chemotaxis clusters**. Positions and orientations of ORFs are indicated by orange and green arrows for flagella and chemotaxis related genes respectfully. Genes which are not flagella related are indicated by their accession number. The map is partial and additional genes such *motA*, *motB *and MCPs are present elsewhere in the genome.

Upon growth on semi-solid surfaces *P. vortex *exhibits at least one form of swarming motility, a flagellum-driven social form of surface locomotion [57-60]. In Figure 5A, we show that propagating *P. vortex *swarms can collectively change direction towards organic matter added to an agar plate, and can even split and reunite when detecting scattered patches of food (see Additional file 1 section I for more details). We previously showed, using flagellar staining and light microcopy that swarming *P. vortex *was peritrichously flagellated (2 to 8 flagella per μm of cell length, 25 to 30 nm wide and > 5 μm long) [14]. These results are in agreement with the dimensions measured by scanning electron microscopy (Figure 5B, C, D). Flagellar motility genes were indentified within the *P. vortex *genome. These genes are located within five different loci, two of which contain the majority of the genes and are 8.4 kb and 27.1 kb long (Figure 6 and Additional file 1 Figure S13).

Social motility could also be powered by the extension and retraction of type IV pili [61,62]. *P. vortex *genome contains several pili-related genes such as *pilZ*, *pilT, flp *pilus assembly protein and prepilin type IV. However, we could not identify all the genes known to be involved in biogenesis and motility of type IV pili [63-65]. Furthermore, the fastest known rate of type IV pili related movement does not exceed 50 μm/min [66,67], whereas, *P. vortex *has an average movement rate of 300 μm/min (data not shown).

Previous studies suggest that the vortices are formed by attractive interaction between swarming cells which can be mediated via attractive chemotactic signaling and/or physical links [3]. The *P. vortex *genome contains several chemotaxis related genes, including the *cheA, cheB, cheC, cheD, cheW *and *cheY*. Many of the chemotaxis genes are located within the large motility loci (Figure 6 and Additional file 1 Figure S14). Additional 16 MCP (methyl-accepting chemotaxis) genes were found in other locations along the genome.

### Sporulation and competence

Formation of spores and uptake of foreign DNA represent an important aspect of bacterial survival strategies. *P. vortex *genome encodes an extensive set of 153 genes responsible for sporulation including cell division, engulfment, cortex and coat synthesis, maturation and germination (Additional file 9). The identified sporulation genes included one of the conserved PFAM domains [53,68], TIGR domains [69], COG categories [42] or KEGG pathways [70] associated with sporulation (Additional file 10).

Although, 9 competence-related genes such as *comEA*, *comer *and *comEC *were identified, they represent only a small portion of the complete competence pathway [71-73]. Additionally, we did not identify homologous genes that belong to the Rap system, which plays an important role in the cell decision-making between sporulation and competence [74,75]. It is therefore possible that the common pathway described for sporulation and competence in other Gram-positive bacteria [76] is different in *P. vortex*.

### Clusters of Multifunctional Enzymes-Secondary Metabolites

**Figure 7:**
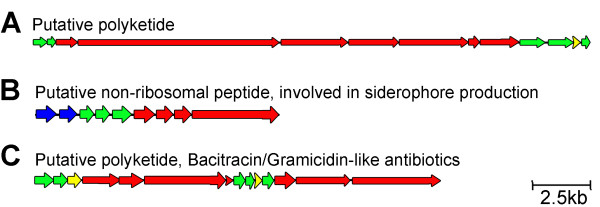
**Clusters of secondary metabolite processing genes identified in the *P. vortex *genome**. Non-ribosomal polyketide synthetase/polyketide synthetase is marked red; transporter genes marked blue; accessory genes marked green; hypothetical genes marked yellow. (A) Putative polyketide synthase gene cluster. (B) Non-ribosomal peptide synthetase gene cluster potentially involved in bacillibactin-like siderophore synthesis. (C) A hybrid gene cluster of polyketide synthase and nonribosomal peptide synthetase involved in bacitracine-like antibiotic synthesis.

Non-ribosomal peptide synthetases (NRPSs) and polyketide synthases (PKSs) are large multi-domain proteins that catalyze the biosynthesis of small molecules with potent biological activity. These molecules, which are mainly produced by bacteria and fungi, often serve as "chemical weapons" against neighboring organisms [77]. Due to their antifungal and antibacterial activities these compounds are also used for medical purposes in the pharmaceutical industry. The PKS genes for a certain polyketide are usually organized in one operon in bacteria and in gene clusters in eukaryotes. We identified 13 PKS, 9 NRPS and 14 PKS genes in the *P. vortex *genome, which were arranged in the following clusters: (i) a 43 kb PKS gene cluster, which is comprised of 13 PKSs involved in polyketide synthesis (Figure 7A); (ii) cluster of 15 kb encoding 9 NRPS, which might be involved in siderophore production (Figure 7B) similar to the bacillibactin siderophore produced by *Bacillus amylolyquefaciens *and *Bacillus subtilis *[78], and (iii) a hybrid 14 PKS/NRPS gene cluster of 23 kb involved in the production of bacitracin-like antibiotics (Figure 7C).

**Figure 8:**
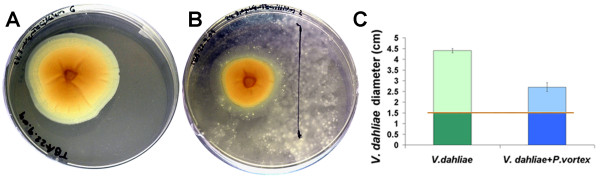
**The antagonistic effect of *P. vortex against Verticillium dahliae***. (A) Control colony of *V. dahliae *fungi grown for 21 days. (B) *V. dahliae *colony was inoculated with *P. vortex *after six days and grown together for additional 15 days. (C) *V. dahliae *diameter (cm) comparison graph for treated (blue) and control (green) colony.

Antagonistic effects of bacteria directed against competing organisms could also result from the enzymatic activity of extracellular degrading enzymes. Seven chitinase and four 1,3-beta glucanase encoding genes were identified in the *P. vortex *genome. These enzymes are involved in degradation of polysaccharide components of the fungal cell wall [79]. Direct tests showed that the *P. vortex *can significantly inhibit the growth of *Verticillium dahlia*, a fungal plant pathogen causing vascular wilt diseases in a broad range of host plants. This plant pathogen is distributed in soil worldwide and is of major threat to agriculture crop production, especially in temperate areas of the world [80]. *P. vortex*, which was inoculated six days after *V. dahliae *inoculation, to allow the establishment of healthy fungal colonies, was able to significantly inhibit the growth of *V. dahliae *(Figure 8A, B). In the first six days, the diameter of *V. dahliae *colonies was identical for all treatment and control plates (~1.5 cm). During 15 days post inoculation of the *P. vortex*, *V. dahliae *colonies grew only ~1.2 cm in diameter, compared to control colonies which grew 2.5 times faster (~2.9 cm in diameter) (Figure 8C).

## Discussion

Whole-genome shotgun pyrosequencing has proved remarkably useful for the large-scale sequencing of bacterial genomes [81-83]. High-quality *de novo *assemblies can be obtained with relatively few errors and gaps when the sequence read coverage redundancy is 15-fold or greater. Closing all the gaps in each genome sequence is time-consuming and costly; therefore, in the near future there will be an excess of draft bacterial sequences versus closed genomes in public databases.

This study presents a *de novo *assembly of the *P. vortex *genome utilizing a hybrid deep-sequencing strategy using a Roche 454 Genome Sequencer (GS 20) and an Illumina Genome Analyzer. The use of the two next-generation leading technologies and the combination of the results into a hybrid assembly overcame the drawbacks of each technology and resulted in longer scaffolds. We demonstrated that the sequence identity between the two methods was 99.88%, reflecting the low error rate of both sequences. The genome sequence, the predicted transcripts and the non-coding RNAs were further validated by hybridization to custom microarray.

Notably, even when using several algorithms and an extremely high coverage, the data could not be assembled into a single sequence. Analysis of the ends of contigs revealed that the unassembled contigs have small repetitive sequences at their ends. The existence of high number of repetitive sequences is a generic obstacle that tempers the ability of the assembly algorithms to generate a single version of the complete genome, and more so when working with short reads. It has been shown that sequence repeats have a functional role that can contribute to genomic plasticity which allows rapid adaptation to environmental changes [48].

*P. vortex *was originally isolated from colonies of *B. subtilis*, soil bacteria commonly found in the rhizosphere [84,85]. The Rhizosphere is characterized by large environmental fluctuations, which act as a selecting force determining the diversity of the microbial community [86-89]. The features identified in the genome of *P. vortex *suggest that these bacteria can lead a successful lifestyle in the highly competitive environment of the rhizosphere as well as serve as an efficient plant beneficial rhizobacteria (PBR). PBR competitively colonize plant roots and can simultaneously act as biofertilizers and as antagonists (biopesticides) of recognized root pathogens [90].

Comparative genomics and comparative network biology are emerging as key tools in understanding of how bacteria respond cooperatively to challenging complex environments. In particular, it was previously suggested that bacteria successful in heterogeneous and competitive environments often contain extensive signal transduction and regulatory networks [25,34,91]. These observations, and the fact that signal transduction networks afford intracellular information processing [36], led to the notion that the number and fraction of signal transduction genes can be used as a measure of the "Bacteria IQ" [34,91]. Detailed comparative genomic analysis revealed that the *P. vortex's *genome and the genome of the Gram-negative, social and predatory bacterium *M. xanthus *[92] have exceptionally high number of TCS genes, supporting the notion that they are required for advanced social behavior.

The *P. vortex *species is marked by its complex spatial organization of the colony, with the bacteria forming different patterns to better cope with the environment [3,4,14,93]. Pattern-formation and self-organization in microbial systems is an intriguing phenomenon that might also provide insights into the evolutionary development of the concerted action of cells in higher organisms [19]. Therefore, sequencing of the *P. vortex *genome paves the way to understanding of regulatory processes involved in cell-cell communication and colonial patterning and more generally, to understanding of cooperative bacterial response to changing environmental conditions. Such information should facilitate increased exploitation of *Paenibacillus *spp. in industrial, agricultural and medical fields, as well as help us comprehend the evolutionary development of multicellular organisms.

## Conclusions

The *P. vortex *genome was sequenced using a hybrid deep-sequencing approach resulting in an estimated genome size of 6.3 Mb. A total of 6,437 ORFs were identified and 73% of them confirmed using specially designed Agilent custom microarray chip. The results of the two sequencing methods were compared resulting in 99.88% sequence identity, reflecting low error rate of both sequences. The use of the two next-generation leading technologies and the combination of the results into a hybrid assembly overcame the drawbacks of each technology and resulted in longer scaffolds.

Comparative genomics analysis with 500 complete bacterial genomes revealed that *P. vortex *has one of the highest number of TCS genes among all the Gram-positive bacteria in the dataset. High numbers of TCS genes were also found in the genome of the social predator *M. xanthus*, supporting the notion that they are required for advanced social behavior. *M. xanthus *serves as an important Gram-negative bacterial model for the study of multicellularity in prokaryotes [94]. Similarly, *P. vortex *may have the potential to provide significant insights on cell-cell interactions, pattern formation and social behavior in Gram-positive bacteria. Additionally, *P. vortex *encodes an extensive set of TFs, transport and defense related genes. These findings suggest that *P. vortex *has a highly developed signal transduction system and that these genes can support traits needed for thriving in heterogeneous, fluctuating and highly competitive environments.

The genome sequence of *P. vortex *provides the basis for understanding of social organization and pattern formation within Gram-positive bacteria. *P. vortex *is the first sequenced *Paenibacillus *species reported to show these properties and this work supports the development of genetic approaches to the study of prokaryotic multicellularity and multi-agent decision making (swarm intelligence). Furthermore, this organism is likely to become a valuable resource for exploitation within biotechnology.

## Methods

### DNA Preparation

*P. vortex *DNA was prepared at two separate times for the 454 and Illumina sequencing runs following the standard Roche and Illumina protocols respectively. *P. vortex was *grown in Luria-Bertani (LB) medium, at 37°C with shaking (200 rpm) over night. DNA was extracted from 2 ml cell culture (10^9^/ml), using Qiagen, DNeasy Blood and Tissue Kit, according to the manufacture's protocol with the following modifications; cells were incubated with Lysosyme for 45 minutes prior extraction. Elution from Qiagen column was performed with 200 μl buffer AE (10 mM Tris-HCl, 0.5 mM EDTA pH 9.0).

### Sequencing

We used a hybrid sequencing approach that incorporates 454 pyrosequencing with Illumina Genome Analyzer. Sequencing by both methods was performed in compliance with manufacturer's instructions Roche and Illumina accordingly.

### Assembly

The 454 reads were assembled using Newbler Assembler [40] version number 1.0.53. To obtain optimized results for the assembly of Illumina short reads we tested several algorithms (Additional file 1 Table S2), but eventually selected Velvet [95]. Velvet's algorithm handled single and paired-end reads and produced contigs with highest sequence identity of 99.88% to those produced by the 454. Algorithms used to assemble short reads are Velvet 0.7.28, Edena 2.1.1 and Euler-SR 1.0. Velvet algorithm was used with parameter hash length of 31, insert length of 250 and minimum contig length 50. Edena algorithm was used with a minimum overlap parameter of 23. The final step included the assembly of the Newbler (454) and Velvet (Illumina) contigs using Minimus 2.0.5 [96].

### Annotation

The DNA sequence was run through JCVI's prokaryotic annotation pipeline (JCVI Annotation Service), which includes gene finding by Glimmer, Blast-extend-repraze (BER) searches, HMM searches, TMHMM searches, SignalP predictions, and automatic annotations from AutoAnnotate. Additionally, the DNA sequence was annotated using NCBI Prokaryotic Genomes Automatic Annotation Pipeline (PGAAP) and the combined annotation was submitted to [GenBank: ADHJ00000000].

### Phylogenetic analysis of 16S

The construction of the phylogenetic tree of 22 taxa was based on 16 S rRNA sequences downloaded in fasta format from DNA Data Bank of Japan (DDBJ) ftp://ftp.ddbj.nig.ac.jp/ddbj_database/16S/. The alignment of the chosen sequences was performed using ClustaX [97] and the construction of the phylegenetic tree using Neighbor-Joining algorithm [98]. The percentage of replicate trees in which the associated taxa clustered together in the bootstrap test (500 replicates) was also calculated [99]. The evolutionary distances were computed using the Maximum Composite Likelihood method [100] utilizing Mega 4 software [101].

### Identification of Two-Component System and Transcription Factor genes

The approach used to identify putative TCS and TF genes utilized HMM (Hidden Markov Model) profiles found in Pfam database of protein families http://pfam.sanger.ac.uk/[53]. TCS genes were identified similarly to that previously described by [102] and [103] and TF genes were identified as described by [55]. The compiled list of Pfam domains that was used to identify TCS and TFs is presented in Additional file 11 and 12 respectfully. Additional methods description is included in Additional file 1 section VII.

### Identification of Transporters and Defense related genes

To identify putative transport and defense related genes we utilized Cluster of Orthologous Groups (COG) profiles [42,43]. The compiled list of COG profiles that were used to identify transport and defense related genes is presented in Additional file 13 and 14 respectfully.

### Combined Score

The combined score was calculated as an average of the standard deviation (stdev) of two-component system, transcription factor, transport and defense genes for the dataset of 500 bacterial genomes. The combined score was calculated both as normalized and non-normalized to genome size.

### Experiment procedure of *P. vortex *effect on *Verticillium dahlia*

*V. dahliae *was grown on trypsin soy agar plates (TSA), at 28°C. A 10 day old stock plate was used to initiate the experiments as follows: A startup slice of 0.5 mm diameter was cut from the colony edge and placed on a fresh TSA plate. The fungal slice was positioned 1 cm away from the center of a 9 mm Petri dish. Plates were incubated untill *V. dahliae *colonies reached 1.5 cm diameter (6 days incubation). At this time-point an overnight *P. vortex *culture, grown in LB, 28°C, with shaking (200 rpm), was inoculated in a 6 cm long line, horizontal to *V. dahliae*. *P. vortex *was positioned 2.5 cm away from the *V. dahliae *colony center. *V. dahliae *colonies without the inoculation of *P. vortex *served as control. All tests were carried out in triplicate.

### Submission to the international collection deposits

Isolate *P. vortex *sp. nov. V453 was deposited at the *Bacillus *Genetic Stock Center (BGSC), Columbus, OH, USA, as strain 31A2^T ^and at the Belgium Coordinated Collection of Microorganisms (BCCM/LMG) as strain LMG 25955.

## List of abbreviations

ORF: Open Reading Frames; TCS: Two-Component System; TF: Transcription Factor; COG: Cluster of Orthologous Groups; GS: Genome Sequencer; GA: Genome Analyzer; HR: Homologous Recombination; RR: Response Regulator; HK: Histidine Kinase; NRPS: Non-Ribosomal Peptide Synthetase; PKS: Polyketide Synthase; PBR: Plant Beneficial Rhizobacteria; HMM: Hidden Markov Model;

## Competing interests

The authors declare that they have no competing interests.

## Authors' contributions

ASM, TO, YH, CI, DLG, DL and EBJ were involved in study design. ASM, TO, YH, CI, IB, DR, EH, RCM, DL, EBJ performed the experiments. IB, RCM and SBZ contributed reagents, materials and analysis tools. ASM, TO, YH, CI, LB, DLE, VG, VK, RCM, DL, EBJ were involved in data analysis. ASM, TO, YH, CI, EBJ wrote the paper. All authors read and approved the final manuscript.

## Supplementary Material

Additional file 1 **Detailed supplementary information**. This file includes additional information on *P. vortex *physiology, comparison of sequencing methods, validation of *P. vortex *annotation and materials and methods. Click here for file

Additional file 2 **Movement of a single vortex, 500× magnification and twice the real speed**. Click here for file

Additional file 3 **Early stage of colony organization including the formation of vortices and moving groups of bacteria**. The magnification is 50× magnification and 60× rate. Click here for file

Additional file 4 **Dynamic imaging of swarming by light microscopy**. Single branch of a swarming culture moving on MH (1.5% w/v) agar is presented. Magnification 400× and 4 times the actual speed. Click here for file

Additional file 5 **Effect of extracellular material derived from plates containing swarming cells on *P. vortex *swarming**. Light microscopy of *P. vortex *moving on MH agar (0.3% w/v), extending into an area where extracellular material derived from washes of swarming cells was delivered by toothpick and allowed to soak into the agar. (A) Cell mass starts to disperse as it approaches the area of the extract. (B) Cell mass has dispersed into area of extract. Click here for file

Additional file 6 **Effect of number of extracellular materials derived from plates containing swarming cells on *P. vortex *swarming**. Light microscopy of *P. vortex *moving on MH agar (0.3% w/v), extending into an area where extracellular material derived from washes of swarming cells. (A) Cell mass is evaluating the gradient and starts move towards the area with the extract. (B) Cell mass starts to disperse as it approaches the area of the extract. (C) Additional cells are moving into this area from further back in the colony. Click here for file

Additional file 7 **A set of 500 complete bacterial genomes of 2-10 Mbp genome size, which were used in the detailed comparative genomic analysis with the *P. vortex *genome**. Click here for file

Additional file 8 **A subset of 50 soil bacterial genomes with genome size 4-8 Mbp (closer to the *P. vortex *genome size), that were used in the comparative genome analysis**. Click here for file

Additional file 9 **List of 153 sporulation genes encoded by the *P. vortex *genome that are responsible for cell division, engulfment, cortex and coat synthesis, maturation and germination processes**. Click here for file

Additional file 10 **List of conserved PFAM domains, TIGR domains, COG categories or KEGG pathways associated with sporulation that were used in identification of sporularion genes in *P. vortex***. Click here for file

Additional file 11 **A compiled list of Pfam domains that was used to identify Two-Component System genes is presented**. Click here for file

Additional file 12 **A compiled list of Pfam domains that was used to identify Transcription Factor genes is presented**. Click here for file

Additional file 13 **A compiled list of COG categories that was used to identify transport related genes is presented**. Click here for file

Additional file 14 **A compiled list of COG categories that was used to identify defense related genes is presented**. Click here for file
