# Supplementary material for: Genome sequence of the pattern forming Paenibacillus vortex bacterium reveals potential for thriving in complex environments
Source: BMC Genomics. 2010 Dec 17;11:710. doi: 10.1186/1471-2164-11-710 (PMC3012674; doi:10.1186/1471-2164-11-710)
Supplement: Additional file 1 — Detailed supplementary information. This file includes additional information on P. vortex physiology, comparison of sequencing methods, validation of P. vortex annotation and materials and methods. [file 1471-2164-11-710-S1.PDF]

Supplemental material to:

Genome sequence of the pattern forming *Paenibacillus vortex* bacterium reveals potential for thriving in complex environments

I. Swarming and Social Behavior of *Paenibacillus vortex*

1.1 Complex modular organization

When grown on semi-solid surfaces, *P. vortex* organize its colonies by generating rotating modules (termed vortices), each consisting of many cells, which are used as building blocks for the colony as a whole [1-17] as is illustrated in Figure S1. Each vortex is composed of many cells that swarm collectively around their common center at about 10 micron/sec (see Movie S1). The vortices vary in size from tens to millions of bacteria, according to their location in the colony. The cells in the vortex replicate, and the vortex expands in size and moves outward as a unit, leaving behind a trail of motile but usually non-replicating cells – the vortex branch. The dynamics of the vortices is quite complicated and includes attraction, repulsion, merging and splitting of vortices. Yet, from this complex, seemingly chaotic dynamics, a colony with complex but non-arbitrary organization emerges. The bacteria in each vortex also have high length variability. Transmission electron microscope (TEM) observations revealed that the elongated cells have several chromosomes.

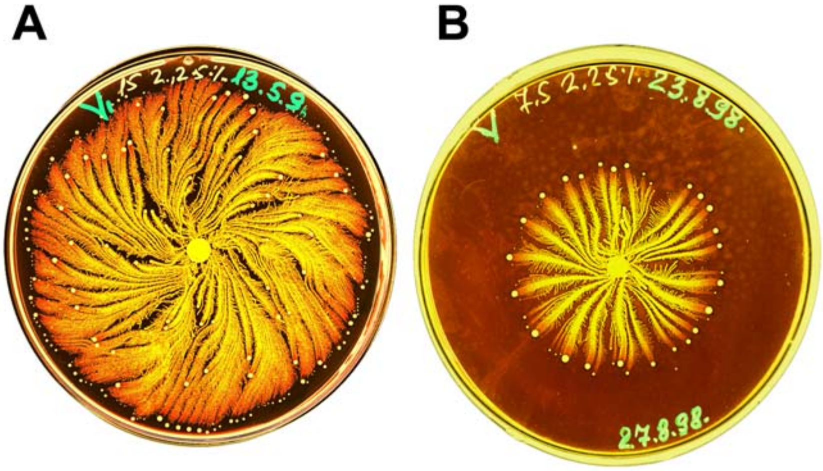

Figure S1: Modular colonial organization of the *Paenibacillus vortex*. A-B shows two examples of the colony organization. The colony growth start from droplet inoculation (the bright spot at the center) of about 10^4-10^5 cells taken after over-night LB growth. The growth conditions on the plates are 15g/l peptone and 2.25% agar concentration in (A) and 7.5g/l peptone and 2.25% agar concentration in (B). The growth time in both cases is 4 days.

Model simulations suggest that the vortices are generated by the action of attractive chemotaxis and possibly physical links such as fibrils between bacteria [1, 2, 6, 8, 9, 11, 12]. It is also suggested that the vortices are "pushed out" in response to repulsive chemotactic agent secreted by bacteria at the center of the colony. Combined with the vortices rotations, the repulsive chemotaxis leads to the curved propagation of the vortices and thus the formation of curved branches [6, 9, 11, 12]. This effect and the structure flexibility are well transparent in the colony patterns shown in Figure S2.

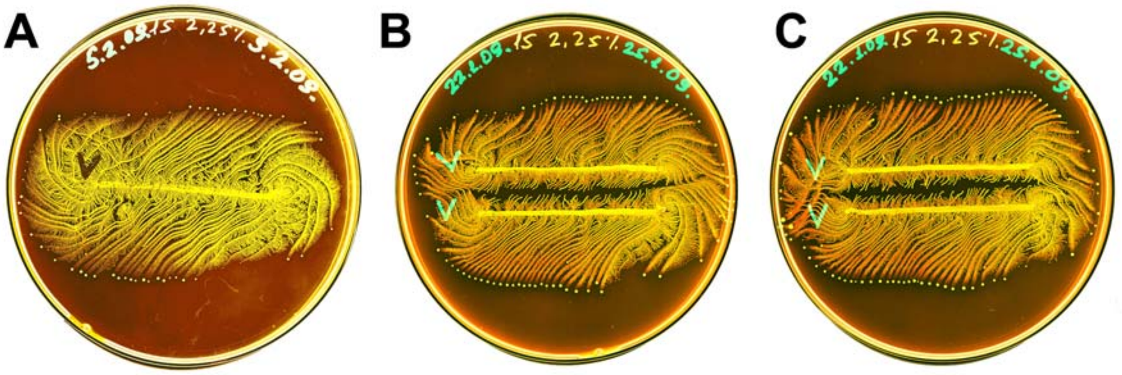

Figure S2: Flexibility of the colony patterns. Colony of *P. vortex* grown on 15g/l peptone and 2.25% agar concentration for 3 days (A) Flexibility of one colony of *P. vortex* inoculated along a parallel straight line (B) Two colonies of *P. vortex* inoculated along two parallel straight lines. Notably, the colonies keep a distance between each other and do not grow on top of each other. (C) The same as B performed at the same time to illustrate the level of reproducibility.

Maintaining the integrity of the individual vortices while they serve as higher-order building blocks of the colony requires advanced communication: each cell has to follow far more complex dynamics, being part of both a specific vortex and the whole colony, so that it can adjust its activities accordingly [12, 15-17]. A greater challenge is posed by the formation of new vortices that emerge in the trail behind a leading vortex. Following initiation signals the non motile cells in the trail start to secrete lubricating fluid and begins to move quite rapidly as a turbulent "biofluid", until an eddy forms and turns into a new vortex (Figure S3) as the cells in the eddy generate strong coupling. The entire process appears to be under advanced communication-based information processing and cooperative control.

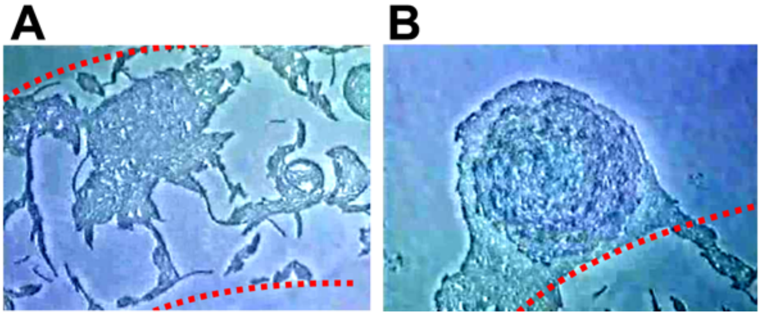

Figure S3: The birth of a new vortex. (A) Trail of cells in vortex that just started to move leading to the formation of a new vortex that can cross the boundaries of the trail (marked by red lines). (B) The mature vortex.

1.2 Adaptability – response to non-lethal levels of antibiotics

In natural habitats bacteria are regularly exposed to non-lethal (sub-inhibitory) levels of antibiotics. The complexity of the colony organization has an important functional role as it affords the bacteria with higher adaptability to cope with environmental stresses [12]. For example, upon encountering antibiotic stress the bacteria re-shape the colony pattern [10, 12, 17, 18] as shown in Figure S4. Response to non-lethal stress of septrin (co-trimoxazole: trimethoprim and sulfamethoxazole) and ampicillin antibiotics is presented (Figure S4 B, C, D). *P. vortex* colonies exposed to multiple stresses septrin and ampicillin, manage to survive in a way that is not fully understood (Figure S4 D).

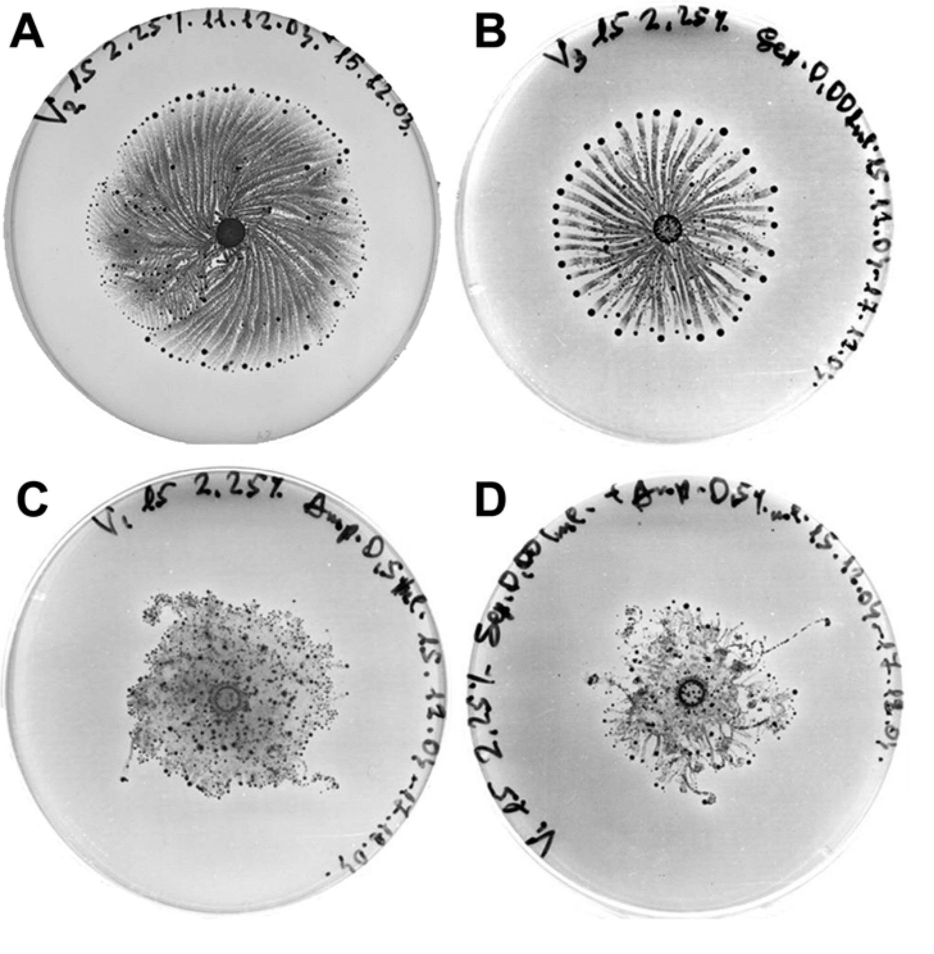

Figure S4: Response to non-lethal levels of antibiotics. Growth conditions are 15g/l peptone and 2.25% agar. (A) Typical colonial development. (B) *P. vortex* colony treated with septrin (co-trimoxazole). (C) A disorganized colonial development in response to ampicillin. (D) Colony exposed to multiple stresses - septrin and ampicillin.

In Figure S5 we show the adaptability of the cells to mitomycin C (MitC) which triggers the SOS system and consequently caused significant filamentation of the individual cells [14]. The effect of MitC on swarming was more extreme on 1.5% (w/v) Mueller Hinton (MH) agar. Real-time imaging revealed that even extremely elongated or curved cells were motile and the drive towards rotation of cell masses was strong (Figure S5 A, B) [14].

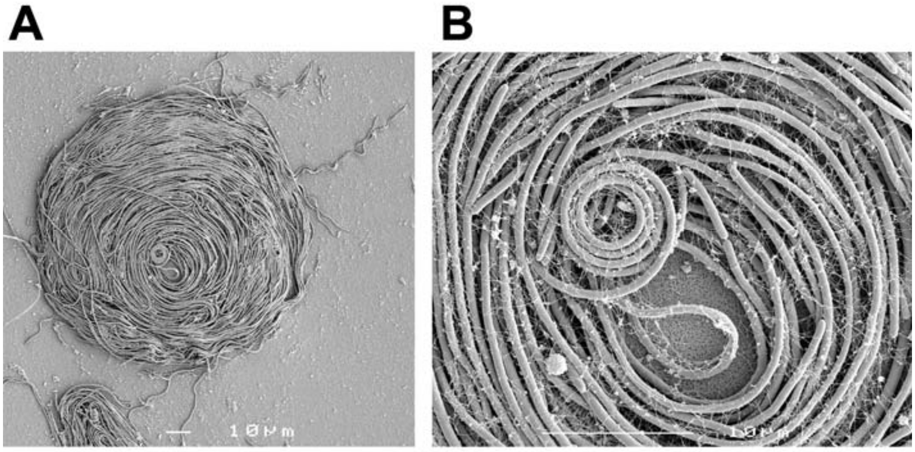

Figure S5: Vortex formation by elongated cells in the presence of MitC. Stills from movies made by transmission light microscopy. (A) SEM of colony on 1.5% MH agar with 0.3 μg/ml MitC showing that the vortex pattern of cells is maintained despite elongation. (B) Center of colony treated with MitC. Scale bar is 10μm when applied to A-B.

1.3 Swarming intelligence

While moving on MH agar, *P. vortex* can form snake-like swarms hundreds of bacteria wide [14]. The swarm can expand very efficiently and collectively change its swarming according to detected chemical cues as shown in Figure S6 and Movies S4-S5. The results illustrate the level of swarming intelligence observed as the bacteria swarm propagates on the surface. As explained in detail in [14], we added to the plate an extracellular material derived from washes of swarming cells which include signaling molecules that the cells secrete. The results suggest, not only the collective change in propagation of the swarm but also a very sophisticated optimization strategy that the bacteria has developed.

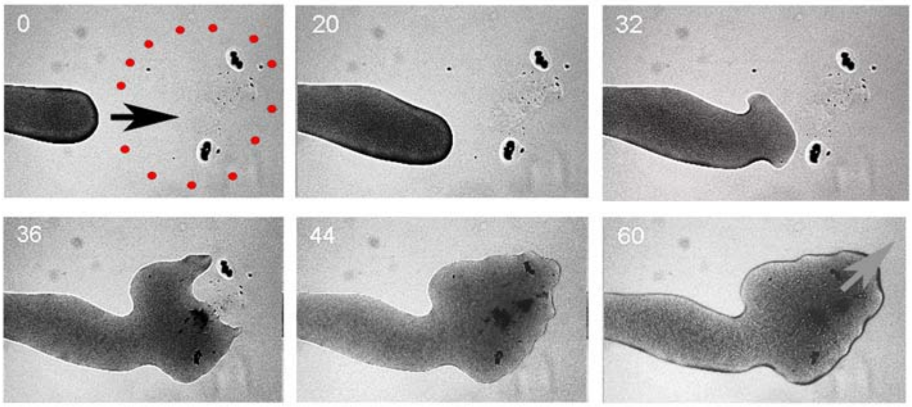

Figure S6: Swarming intelligence of the *P. vortex* bacteria. Snapshots from a video clip of a branch of the *P. vortex* colony moving on 0.3% w/v Mueller-Hinton agar (M-H agar) (x50 magnification). The branch is extending into an area with extracellular material. The time frame capture is noted in seconds. Starting (t=0-20): Area of extract outlined in red dots with direction of cell mass elongation shown by the black arrow. Stage t=32: cell mass starts to disperse as it contacts the area of the extract. Stage t=44: Cell mass has dispersed into area of extract. Stage t=60: Additional cells are moving into this area from the back of the colony; the cell mass is growing in volume and extends in the direction of the grey arrow.

II. Comparison of Sequencing Methods and Assembly Algorithms

The *P. vortex* reads obtained by the two high-throughput sequencing technologies the Roche 454 Genome Sequencer (GS 20) [19] and the Ilumina Genome Analyzer (GA) [20] were assembled using various algorithms and compared to assess the drawback of each technology. Using the Roche 454 and the Illumina GA technologies, 19x coverage of single reads, and 270x total average coverage of single and paired-end mapped reads was produced respectfully. The reads from each technology were first assembled separately and then joined into a hybrid assembly to improve scaffold size and quality.

To obtain optimized results we tested several short reads algorithms (Table S1), but eventually selected Velvet [21] to process Illumina's short reads. Velvet's algorithm easily handled single and paired-end reads and produced contigs with the highest sequence identity (99.88%) to those produced by the 454. The hybrid assembly, contains 56 scaffolds totaled 6,385,925bp with N50 scaffold size of 213,399bp and the largest scaffold of 699,613bp. Notably, the contigs from the two technologies could be joined easily as no miss-assemblies were detected between the two sets of contigs.

Input Reads	Assembler	No. of Contigs >500bp	Largest Contig	Average contig length	Assembly length (bp)
Illumina Single and Paired-end Reads	Velvet	224	229,208	28,339	6,348,003
Illumina Single Reads	Velvet	1220	40,778	5,175	6,313,642
	Edena	618	75,984	10,237	6,326,849
	Euler-SR	546	121,625	11,607	6,337,795
454 Single Reads	Newbler	227	168,569	27,737	6,379,633
Illumuna and 454 Contigs	Minimus	56	699,613	102,998	6,385,925

Table S1: Statistics of the different assemblies generated by various short read algorithms utilized in the project. Newbler was used to process the 454 reads. Velvet, Edena and Euler were used to process the Illumina data. The final hybrid assembly was generated by merging and assembling the Newbler and Velvet contigs.

We analyzed the distance between the contigs which were assembled by each method and later were united into larger contigs using the hybrid assembly. The average distance of the 454 contigs that was closed retrospectively by the hybrid assembly was -5bp and of the Illumina contigs -10bp (Table S2). The sequence identity between the two methods was 99.81%.

	Newbler (454 reads)		***Velvet (Illumina Single and Paired-end reads)***	
Uncovered Region (bp)	890		4,500	
Distance between contigs Min, Max (bp)	-133	74	-86	296
Mean distance between contigs (bp)	-5		-10	
Coverage of the hybrid assembly	99.93%		99.81%	
Sequence identity between Velvet and Newbler	99.88%			

Table S2: Performance comparison between Newbler and Velvet algorithms.

A comparison of the 454 and Illumina contigs vs. the hybrid contigs is presented (Figure S6). Schematic representation of the hybrid scaffold [0002] (colored red), generated using 454 contigs (colored green) and Illumina contigs (colored blue) is presented (Figure S6A). Contig length distribution of each of the methods indicates that the 454 and the Illumina contigs are similar in their size compared to the hybrid contigs which are much longer (Figure S6B). In Figure S6D, contigs in all sizes were ordered by the ranks and plotted by log scale of the contig size. This demonstrates that the hybrid assembly comprise of a longer contigs compared to the contigs produced by each of the technologies. Additionally, inter-contig length distribution analyzed post-hoc the hybrid assembly is presented (Figure S6C).

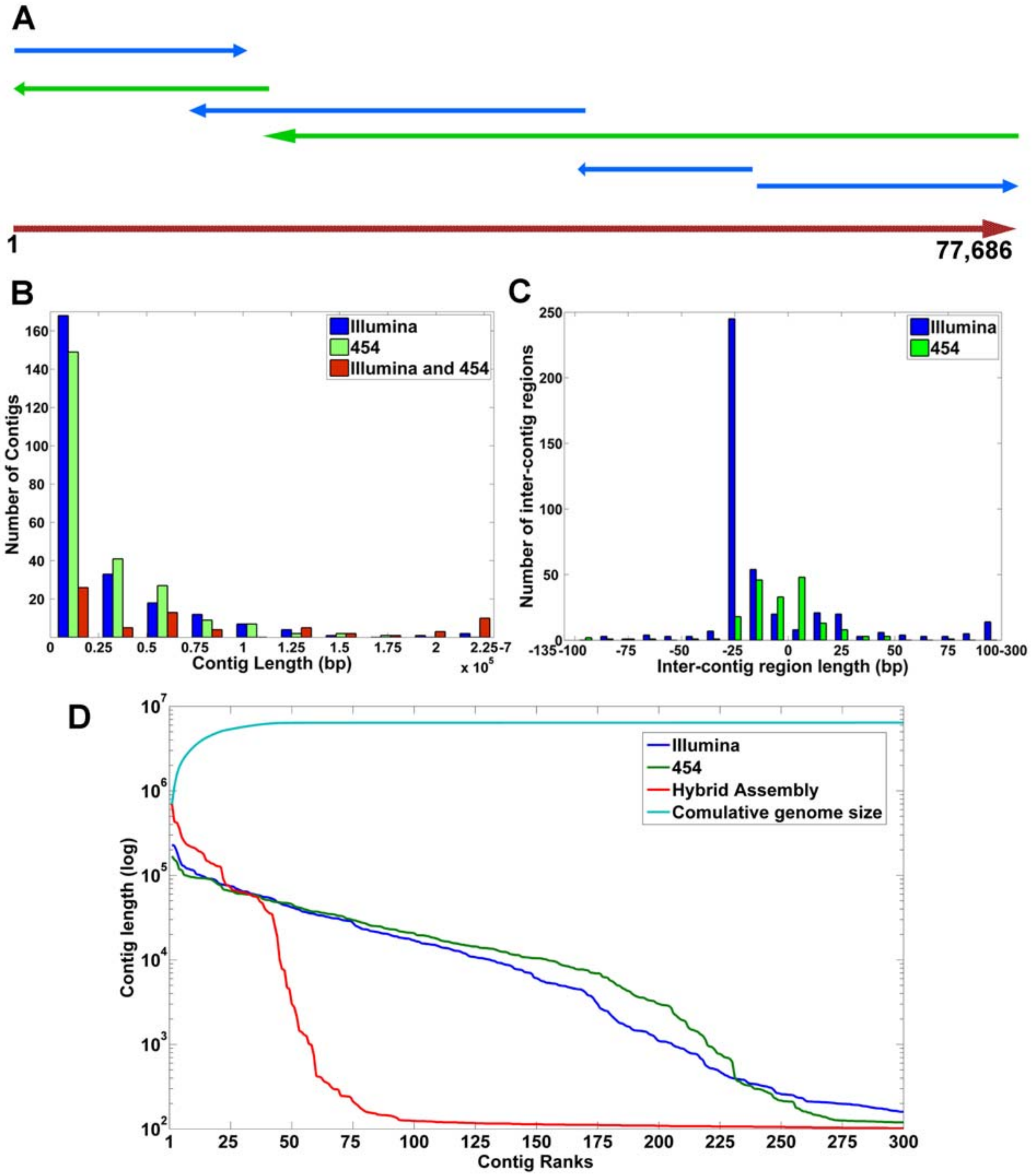

Figure S6: A comparison of the 454 and Illumina sequencing results versus the hybrid assembly. *(A)* Schematic representation of hybrid scaffold [0002] generated using contigs of 454 technology (colored in green) and the Illumina Genome analyzer (colored in blue). The consensus sequence of the scaffold[0002] assembled by the two methods is colored in red. *(B-D)* Results of the statistic analysis of contigs generated by the two methods. *(B)* Contig length distribution. *(C)* Inter-contig length distribution analyzed post-hoc the hybrid assembly. (*D*) Contig length comparison between the two methods and the hybrid assembly is presented. The first 300 contigs (all sizes) were plotted. The cumulative genome size is marked in cyan.

III. Scaffold Ordering

When ordering and assembling the *P. vortex* contigs into a pseudo-molecule, we identified the *Geobacillus* sp. Y412MC10 (Refseq: NC_013406) as the closest bacteria with a complete genome. This identification was based on phylogenetic analysis of 16S placing the *Geobacillus* sp. Y412MC10 within the *P. vortex* clade (Figure 2A). Further supported by BLASTn comparison results (Figure S7 A) and by genomic clustering of Cluster of Ortholog Genes (COG) profiles (Figure 2B). Comparison of the *P. vortex* genome vs. the *Geobacillus* sp. Y412MC10, revealed that 2/3 of the *P. vortex* genome could be matched to *Geobacillus* sp. Y412MC10 with an average sequence identity of 86.69% over a mean alignment length of 783bp. The similarity between the two genomes was significantly higher than the similarity between the *P. vortex* and the *B. subtilis* genomes (Figure S7 A).

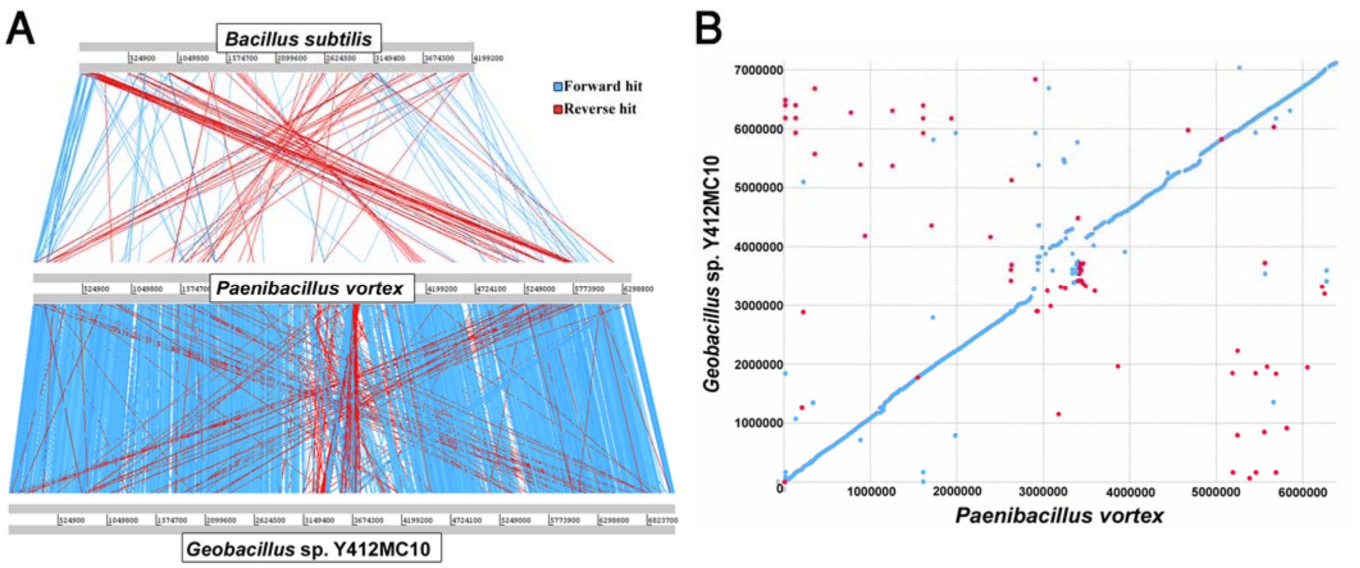

Figure S7: Genome comparison between *P. vortex* and *Geobacillus* sp. Y412MC10. (A) Visualization of the BLASTn results between *P. vortex*, *Geobacillus* sp. Y412MC10 and *B. subtilis* sequence. Forward hit marked in blue, reversed fit marked in red. (B) Dot plot representation of the alignment of the *P. vortex* and the *Geobacillus* sp. Y412MC10 sequences. Forward hits are marked in red and reverse hits in blue.

We utilized MUMmer 3.20 [22] to produce a dot plot of the alignment of the *P. vortex* and the *Geobacillus* sp. Y412MC10 sequences (Figure S7 B). MUMmer efficiently locates maximal unique matches between two sequences using a suffix tree data structure. A line of dots with slope = 1 represents an undisturbed segment of conservation between the two sequences, while a line of slope = -1 represents an inverted segment of conservation between the two sequences. MUMmer was used to find all the 30 base pair maximal exact matches between the two sequences. A strong

diagonal between the *P. vortex* and the *Geobacillus* sp. Y412MC10 genome sequences was observed indicating high conservation between the two genomes.

In addition, the COG profiles of several species were analyzed to further understand the relation of the *Geobacillus* sp. Y412MC10 genome in the genetic context. We performed genome clustering based on COG profiles and presented the results using Pearson correlation matrix. Abundance profile vector of 4873 COGs was calculated for 25 different bacterial species representing various taxons (Table S3).

	COG0001	COG0002	COG0003	COG0004	COG0005
***Bacillus amyloliquefaciens* FZB42**	3	1	0	1	1
***Bacillus anthracis* Tsiankovskii-I**	2	1	2	1	1
***Bacillus cereus* 03BB102**	2	1	1	1	1
***Bacillus halodurans* C-125**	3	1	1	1	2
***Bacillus mycoides* DSM 2048**	2	1	1	1	1
***Bacillus pumilus* ATCC 7061**	2	1	0	1	1
***Bacillus subtilis* 168**	2	1	0	1	1
***Geobacillus kaustophilus* HTA426**	2	1	0	1	1
***Geobacillus* sp. G11MC16**	2	1	0	1	1
***Geobacillus* sp. WCH70**	3	1	0	0	1
***Geobacillus* sp. Y412MC10**	2	1	0	1	1
***Geobacillus* sp. Y412MC61**	2	1	0	2	1
***Geobacillus thermodenitrificans* NG80-2**	2	1	0	1	1
***Myxococcus xanthus* DK 1622**	3	1	6	1	2
***Paenibacillus dendritiformis* C451**	2	1	0	1	1
***Paenibacillus larvae larvae* BRL-230010**	3	0	0	0	2
***Paenibacillus* sp. JDR-2**	4	2	0	3	1
Paenibacillus vortex	2	1	0	1	1
***Pseudomonas aeruginosa* 2192**	4	1	0	2	1
***Pseudomonas fluorescens* PfO-1**	1	1	0	2	1
***Pseudomonas syringae* pv. tomato DC3000**	1	1	0	2	1
***Sorangium cellulosum* So ce 56**	2	1	2	2	2
***Vibrio cholerae* 623-39**	1	1	0	1	0
***Vibrio fischeri* ES114**	1	1	0	1	0
***Vibrio splendidus* 12B01**	1	1	0	1	0

Table S3: Example of abundance profile vector of 4873 COGs for 25 different bacterial species is presented. Pearson correlation was further applied on the matrix and cluster arrangement illustrated using a dendrogram.

The computed Pearson correlation matrix was ordered using the dendrogram clustering algorithm to identify clusters in the correlation organization. The analysis revealed that the *Geobacillus sp.* Y412MC10 is clustered with *Paenibacillus* species and not with the rest of the *Geobacillus* species (Figure 2B). This result implies that the *Geobacillus* sp. Y412MC10 bacteria might belong to the *Paenibacillus* taxon. The analysis also revealed that *Paenibacillus larvae* is not part of the *Paenibacillus* cluster of soil bacteria (*P. vortex*, *P. dendritiformis and Paenibacillus.* sp. JDR-2).

IV. Validation of the *P. vortex* genome and annotation using custom microarray chip

The *P. vortex* genome annotation was validated using a specially designed Agilent custom chip microarray. A total of 105,000 probes (60 mers) representing both the predicted ORFs and the genomic sequence, including the intergenic regions, were designed (Figure S8).

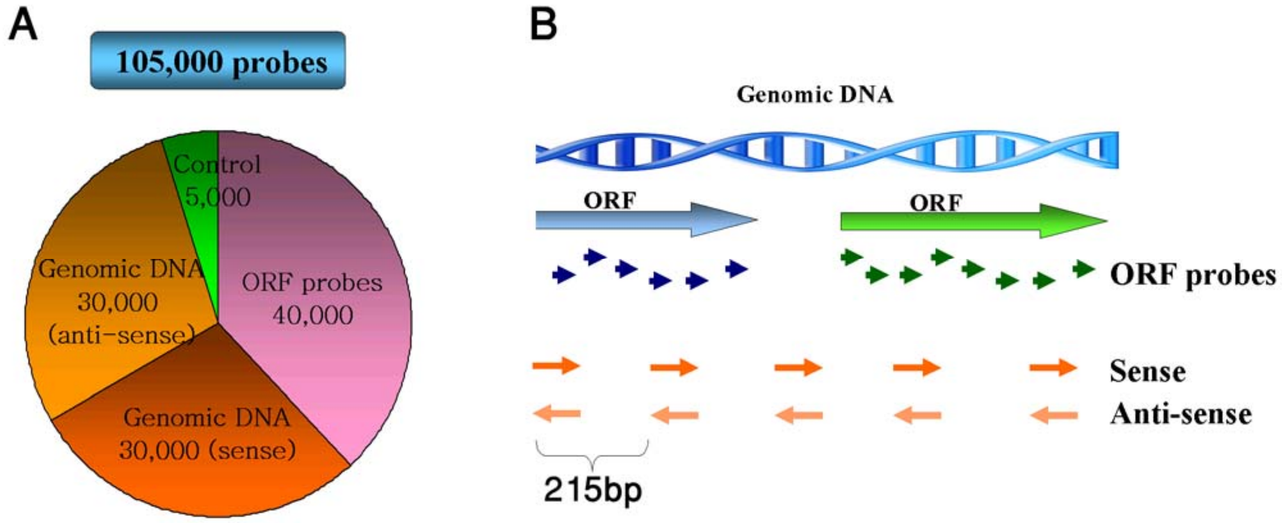

Figure S8: Schematic representation of the probe design process. (A) Source distribution of the 105,000 probes selected for the chip. (B) Schematic representation of how the probes were selected. Average of eight probes per each predicted ORF was designed, resulting in total 40,000 probes. The genome sequence was sampled every 215bp in sense and anti-sense direction, therefore including the coding and the non-coding regions. Thus, total 60,000 probes were designed to represent the genomic sequence. Additional 5,000 control probes were placed on the chip.

The RNA was extracted from a pool of stress conditions such as heat shock, oxidative shock and exposure to various antibiotics, and from non-stress conditions (bacteria grown on peptone, LB and Muler Hinton agar) and labeled with two different colors Cy3 and Cy5 respectfully (Figure S9). Hybridization of the pooled RNA from stress vs. non-stress conditions highlighted 4,701 (73%) of the predicted ORFs. The remaining 1,736 (27%) ORFs were not expressed under the tested experimental conditions. Out of those, 1,064 ORFs have an assigned putative function and 672 are hypothetical. This result can imply that some of the ORFs were not transcribed under the tested growth conditions and some might be an artifact of an automatic annotation (e.g. short hypothetical genes).

Hybridization of the genomic DNA validated 91,324 probes (88%) of the total designed probes and no missed regions were found. Hybridization of predicted 73 non-coding RNAs located within the intergenic regions, ratified 43 (58%). Example of the highlighted probes covering the predicted 16S within the intergenic region is shown in (Figure S10).

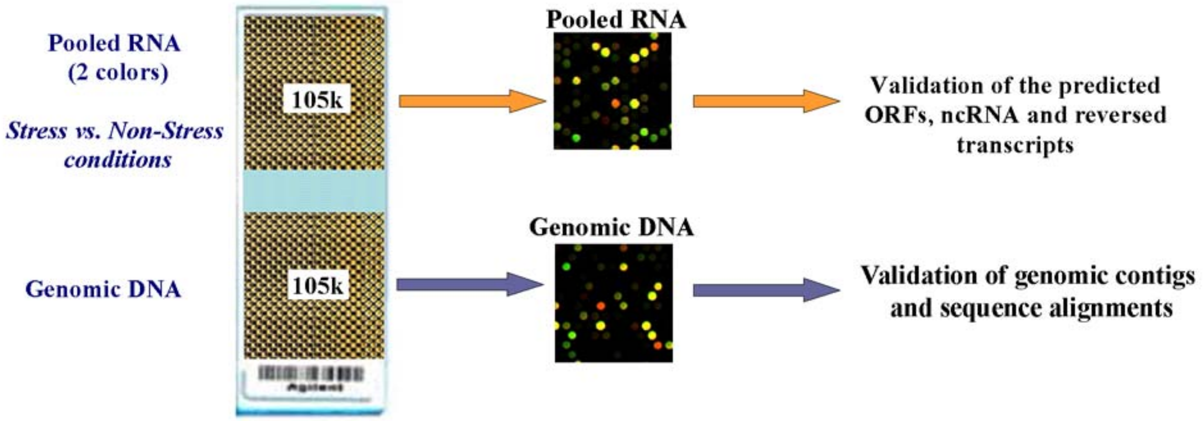

Figure S9: Workflow of the microarray experiment. Pooled RNA from stress and non-stress conditions was extracted and labeled with two different colors cy3 and cy5 respectfully. The RNA, which was hybridized on the first chip containing the 105k probes, validated 4,701 (73%) of the predicted ORFs. The genomic DNA, which was hybridized on the second chip, validated 91,324 probes (88%) of the total designed 105K probes, and ratified 43 (58%) ncRNAs out of total 73 predicted.

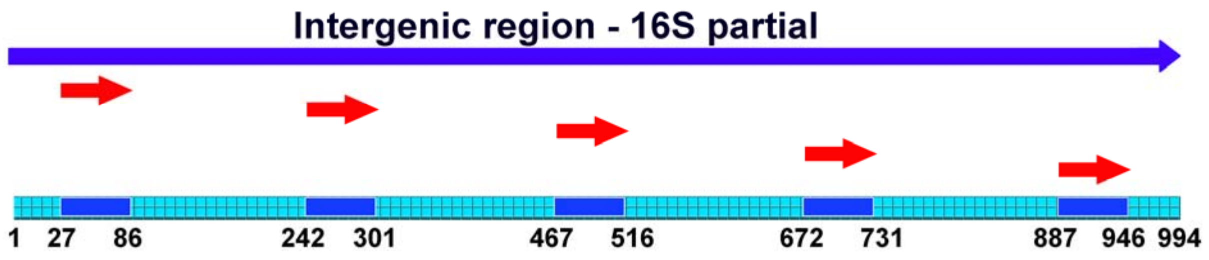

Figure S10: Evidence for 16S RNA expression using the Agilent microarray hybridization. The probes, marked in red, hybridized to DNA and pooled RNA and uniquely map to the intergenic regions of the 16S gene.

V. Comparative Genomics

Detailed comparative analysis was performed between the *P. vortex* genome and a set of 500 complete bacterial genomes of 2-10Mbp genome size. The comparison was done with regard to the two-component system (TCS) genes, transporter related genes, transcription factors (TFs) and genes associated with defense mechanisms.

Two-component system (TCS): We identified a total of 210 TSC related genes in the *P. vortex* genome; 103 response regulators (RRs), 97 histidine-kinases (HKs) and 10 hybrid kinases. Analysis of subset of 261 genomes with genome size of 4-8Mbp, showed that the average number of TCS genes is 85±34 which is significantly lower than the number of TCS genes found in *P. vortex* (Figure 4A).

Transporters: A total 700 transport related genes were identified in the *P. vortex* genome (Figure 4B). Among the 50 soil bacteria, *Paenibacillus* sp. JDR-2 and *P. vortex* have the highest number of 753 and 700 transport encoding genes respectfully.

Transcription Factors (TFs): We found a general linear dependence between the TFs and the genome size (Figure 4C). A total of 411 Transcription Factors (TFs) were identified in *P. vortex* genome, which placed it at the upper 5% of the 500 bacteria set (Figure 4C). This number is considerably higher than the average 208±92 TFs among the subset of 261 genomes with size 4-8Mbp sizes

Defense Mechanism: The *P. vortex* genome harbors 138 genes associated with defense mechanisms which employ resistance to various substances such as antibiotics, copper, aluminium, arsenic and toxic anions (Figure 4D). Apart from *Streptomyces griseus* NBRC 13350 (8.54Mbp), *P. vortex* possesses the highest number of defense related genes among the 500 analyzed genomes. Additionally, *P. vortex* has the highest number of these genes compared to the subset of 261 genomes with 4-8Mbp genome size (the average for this subset is 60±20).

Studies presenting comparative genome analysis commonly use values of relative gene number or fraction of gene numbers (the number of genes divided by the total number of genes in the genome) instead of taking the absolute (unprocessed) gene numbers. Figure S11 presents comparative analysis of TCS, TF, transport and defense genes with regards to the fraction of

genes relative to bacteria genome size (Mbp). Our results show that similarly to the absolute gene numbers, the relative gene numbers of the tested categories in *P. vortex* genome is also significantly higher compared to the rest of the 500 genomes. Nevertheless, we chose to use the absolute values of the genes (Figure S12) as a measure of bacteria's ability to survive in heterogeneous environments. This stems from the fact that social behavior and the ability to cope with fluctuating environments largely depend on the timely response of bacteria to chemical stimuli released in the environment. Such timely response is dependent on the number (and not their fraction compared to total proteins) of participating proteins that sense, utilize and/or detoxify specific substances.

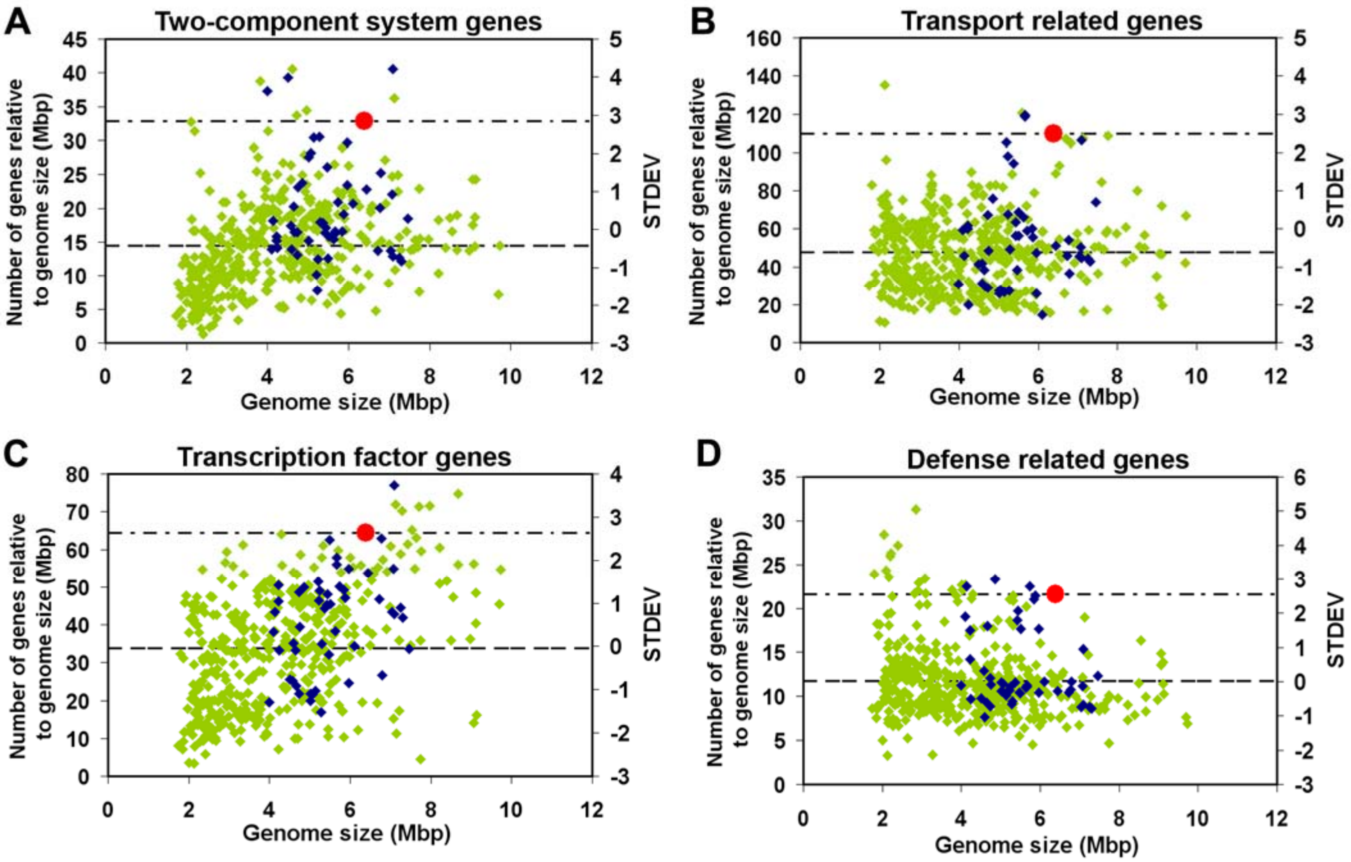

Figure S11: Number of genes normalized to the genome size (Mbp) for 500 bacterial genomes as a function of genome size is presented. Gene number for 50 soil bacterial genomes sized between 4-8Mbp marked in blue and the rest of the genomes marked in green. *P. vortex* marked in red and its value as dotted line. STDEV for each of the graphs is presented on the right side of the axis. The mean value is presented in the dashed line. *(A)* Two-component system (TCS) genes plot is presented. *(B)* Transport related genes are presented. *(C)* Transcription factor genes *(D)* Defense related genes.

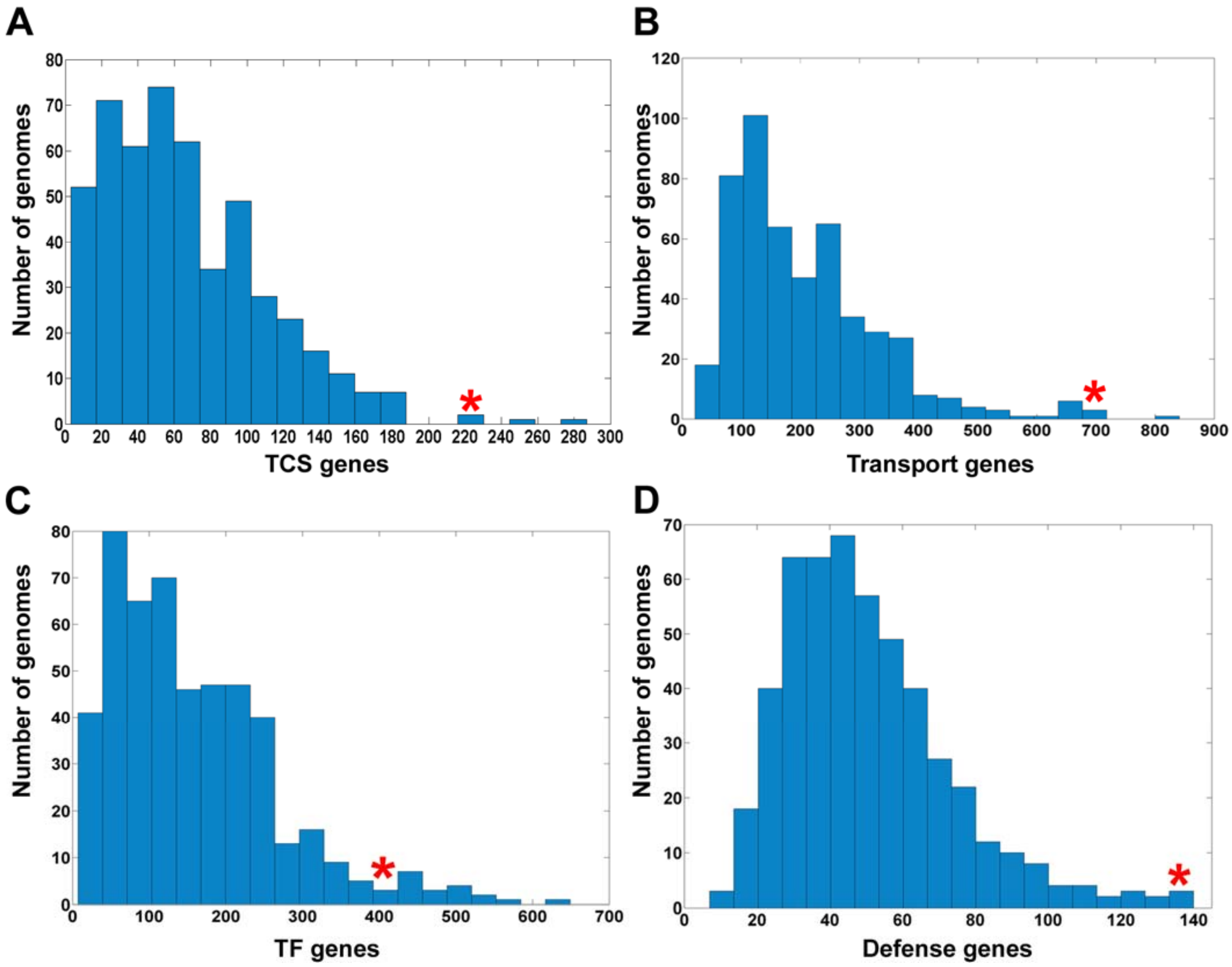

Figure S12: Score Distributions - histograms of the bacteria genomes according to the absolute number of genes for the data set of 500 bacteria. Value of the *P. vortex* in each of the categories is marked in red star and located above the relevant bin. *(A)* Two-component system genes distribution. *(B)* Transport related genes distribution. *(C)* Transcription factor genes distribution. *(D)* Defense related genes distribution.

VI. Flagella Motility and Chemotaxis

Flagella Assembly: *P. vortex* harbors many genes encoding the flagellar assembly (marked in red in Figure S13) which are located within five different loci, two of which 8.4kb and 27.1kb long contain the majority of the genes. The flagellar assembly pathway was redrawn from KEGG (Kyoto Encyclopedia of Genes and Genomes) [23].

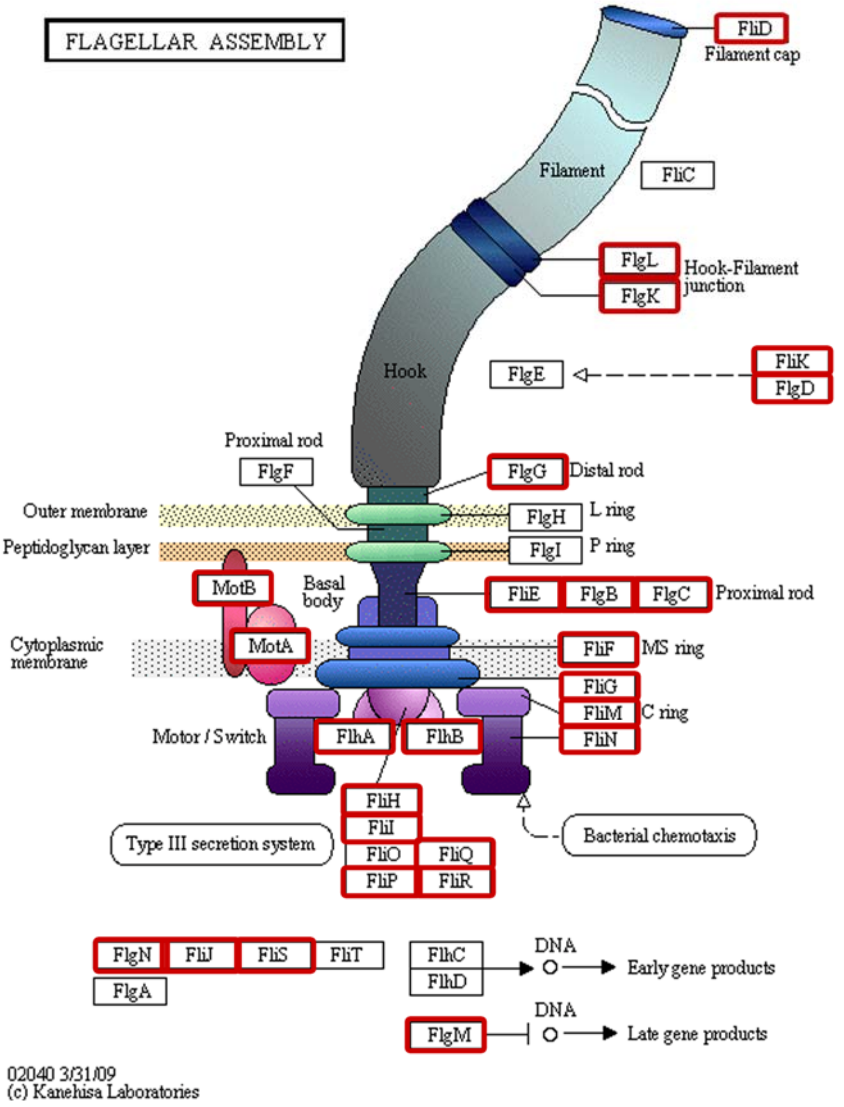

Figure S13: Flagellar assembly pathway as presented in KEGG. Genes encoded in *P. vortex* are marked in red.

Chemotaxis: The *P. vortex* genome encodes several chemotaxis related genes, including the *cheA, cheB, cheC, cheD, cheW* and *cheY.* These chemotaxis genes are located within large motility loci and marked red in Figure S14. Additional 16 MCP (methyl-accepting chemotaxis) genes were found in other locations along the genome. The bacterial chemotaxis pathway was redrawn from KEGG.

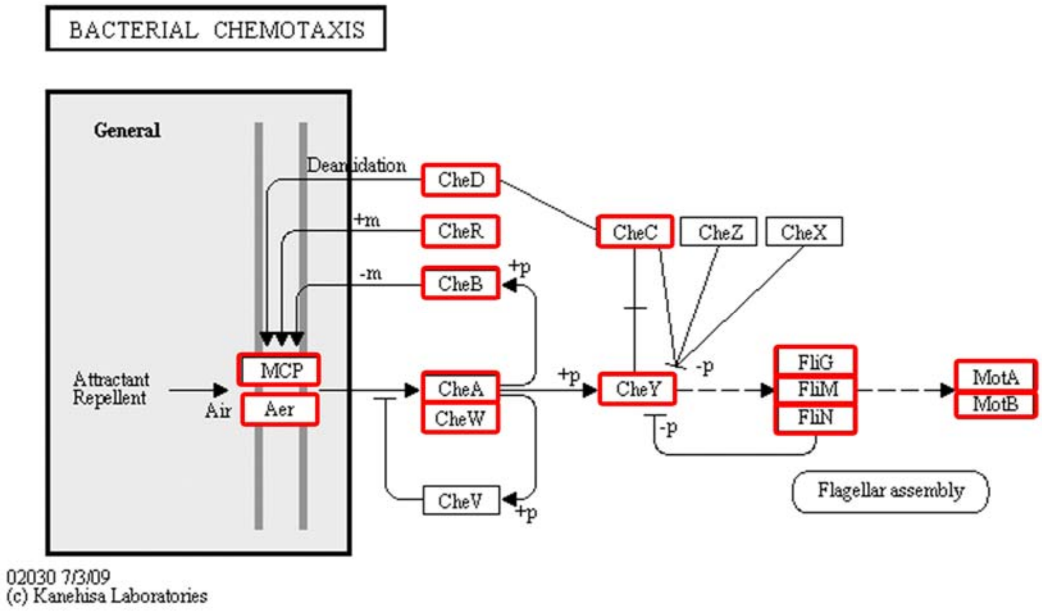

Figure S14: Bacterial chemotaxis pathway as presented in KEGG. Chemotaxis genes coded by *P. vortex* are marked in red.

VII. Methods

Identification of Two-Component System genes: The approach used to identify putative Histidien Kinases (HKs) and Response Regulators (RRs) from the complete genome sequences of *P. vortex* was similar to that described previously by [24] and [25]. For the identification of TCS genes we used HMM (Hidden Markov model) profiles found in Pfam database of protein families (http://pfam.sanger.ac.uk/) [26]. The recognition of TCS genes was based on the following transmitter domains (HisKA - PF00512, HisKA_2 - PF07568, HisKA_3 - PF07730, HWE_HK - PF07536, Hpt - PF01627, His_kinase -PF06580) and a receiver domain (REC - PF00072) found in Pfam database. Putative TCS genes were then classified as response regulators (RRs; proteins encoding receiver domains but no transmitter domains), histidine kinases (HKs; transmitter domains but no receiver domains), hybrid kinases (HYs; transmitter and receiver domains) or phosphotransfer proteins [Ps; Hpt or HisKA domains but no catalytic domain histidine ATPase (HATPase - PF02518) or receiver domains]. We applied the method described above to identify TCS genes in 500 complete bacterial genomes using the Integrated Microbial Genomes (IMG) system [27]. Structural classification of the *P. vortex* RRs was preformed according to scheme previously proposed by M.Y. Galperin [28].

Gene organization of TCS genes was determined using a classification scheme based on proximity to other TCS genes. If a TCS gene was separated from other TCS genes by >5000bp it was considered to be orphan; paired TCSs were defined as two TCS genes on the same strand of DNA and separated by <100bp, encoding a total of one transmitter and one receiver domain. Any other gene organization was defined as complex, including gene clusters encoding multiple TCS genes containing more than one transmitter and/or more than one receiver domain. Orphan hybrid kinases were considered to be orphans regardless of the number of transmitter and receiver domains they encoded. TCS neighbor genes were identified by inspecting the two upstream and downstream genes of the HK and RR genes.

Identification of Transcription Factors: The *P. vortex* Transcription Factors genes were identified as described by [29]. The prediction method identifies sequence-specific DNA-binding transcription factors through homology using Hidden Markov Models (HMMs) of domains. The collection of HMMs was taken from Pfam database version 18.0, which was manually compiled to include models that exclusively detect transcription factors that specifically recognize DNA sequences (Additional file 12). It does not include for example, basal transcription factors or chromatin-associated proteins. A protein was considered to be a TF if it has one of the 146

sequence specific DNA binding Pfam domains. We applied the method described above to identify TF genes in 500 complete bacterial genomes using the Integrated Microbial Genomes (IMG) system [27].

Identification of repetitive sequences: Global repeats were searched using Blast of the *P. vortex* genome vs. itself, results above e-value higher than E-05 were included. Inverted repeats were searched using Inverted Repeats Database (IRDB) version 2.2 (https://tandem.bu.edu/cgi-bin/irdb/irdb.exe?redirect=yes&taskid=0), tandem repeats were searched using Tandem Repeat Database (TRDB) version 2.3 http://tandem.bu.edu/cgi-bin/trdb/trdb.exe [30] and both were run default parameters.

Visualization of the genome properties: The properties of the *P. vortex* genome including scaffolds, genes, sequencing coverage and repetitive sequences were visualized using Circos [31]. Circos is an effective tool for displaying variation in genome structure and any other kind of positional relationships between genomic intervals. Circos uses a circular ideogram layout to facilitate the display of relationships between pairs of positions by the use of ribbons, which encode the position, size, and orientation of related genomic elements.

Supplementary References

1. Ben-Jacob E: **From snowflake formation to growth of bacterial colonies II: Cooperative formation of complex colonial patterns.** *Contem Phys* 1997, **38:**205 - 241.
2. Ben-Jacob E, Cohen I: **Cooperative formation of bacterial patterns.** In *Bacteria as Multicellular Organisms* Edited by Shapiro JA, Dworkin M. New York: Oxford University Press; 1997: 394-416
3. Ben-Jacob E, Cohen I, Shochet O, Aranson I, Levine H, Tsimring L: **Complex bacterial patterns.** *Nature* 1995, **373:**566-567.
4. Ben-Jacob E, Schochet O, Tenenbaum A, Cohen I, Czirok A, Vicsek T: **Generic modelling of cooperative growth patterns in bacterial colonies.** *Nature* 1994, **368:**46-49.
5. Ben-Jacob E, Shochet O, Tenenbaum A, Avidan O: **Evolution of complexity during growth of bacterial colonies.** In *NATO Advanced Research Workshop; Santa Fe, USA.* Edited by Cladis PE, Palffy-Muhorey P. Addison-Wesley Publishing Company; 1995: 619-633.
6. Ben-Jacob E, Cohen I, Czirók A, Vicsek T, Gutnick DL: **Chemomodulation of cellular movement, collective formation of vortices by swarming bacteria, and colonial development.** *Physica A* 1997, **238:**181-197.
7. Ben-Jacob E, Cohen I, Gutnick DL: **Cooperative organization of bacterial colonies: from genotype to morphotype.** *Annu Rev Microbiol* 1998, **52:**779-806.
8. Cohen I, Czirok A, Ben-Jacob E: **Chemotactic-based adaptive self-organization during colonial development.** *Physica A* 1996, **233:**678-698.
9. Czirok A, Ben-Jacob E, Cohen II, Vicsek T: **Formation of complex bacterial colonies via self-generated vortices.** *Phys Rev E Stat Phys Plasmas Fluids Relat Interdiscip Topics* 1996, **54:**1791-1801.
10. Ben-Jacob E, Cohen I, Golding I, Gutnick DL, Tcherpakov M, Helbing D, Ron IG: **Bacterial cooperative organization under antibiotic stress.** *Physica A* 2000, **282:**247-282.
11. Ben-Jacob E, Cohen I, Levine H: **Cooperative self-organization of microorganisms.** *Adv Phys* 2000, **49:**395-554.
12. Ben-Jacob E: **Bacterial self-organization: co-enhancement of complexification and adaptability in a dynamic environment.** *Phil Trans R Soc Lond A* 2003, **361:**1283-1312.

13. Ben-Jacob E, Levine H: **Self-engineering capabilities of bacteria.** *J R Soc Interface* 2005, **3:**197-214.

14. Ingham CJ, Ben-Jacob E: **Swarming and complex pattern formation in *Paenibacillus vortex* studied by imaging and tracking cells.** *BMC Microbiol* 2008, **8:**36.

15. Ben-Jacob E: **Social behavior of bacteria: from physics to complex organization.** *Eur Phys J B* 2008, **65:**315-322.

16. Ben-Jacob E: **Bacterial Complexity: More is Different on all Levels.** In *Systems Biology: The Challenge of Complexity*. Edited by Nakanishi S, Kageyama R, Watanabe D. Tokyo: Springer; 2009: 25-35

17. Ben-Jacob E, Becker I, Shapira Y, Levine H: **Bacterial linguistic communication and social intelligence.** *Trends Microbiol* 2004, **12:**366-372.

18. Ben-Jacob E, Aharonov Y, Shapira Y: **Bacteria harnessing complexity.** *Biofilms* 2004, **4:**239-263.

19. Margulies M, Egholm M, Altman WE, Attiya S, Bader JS, Bemben LA, Berka J, Braverman MS, Chen YJ, Chen Z, et al: **Genome sequencing in microfabricated high-density picolitre reactors.** *Nature* 2005, **437:**376-380.

20. Bentley DR, Balasubramanian S, Swerdlow HP, Smith GP, Milton J, Brown CG, Hall KP, Evers DJ, Barnes CL, Bignell HR, et al: **Accurate whole human genome sequencing using reversible terminator chemistry.** *Nature* 2008, **456:**53-59.

21. Zerbino DR, Birney E: **Velvet: Algorithms for de novo short read assembly using de Bruijn graphs.** *Genome Res* 2008, **18:**821-829.

22. Kurtz S, Phillippy A, Delcher AL, Smoot M, Shumway M, Antonescu C, Salzberg SL: **Versatile and open software for comparing large genomes.** *Genome Biol* 2004, **5:**R12.

23. Kanehisa M, Goto S, Hattori M, Aoki-Kinoshita KF, Itoh M, Kawashima S, Katayama T, Araki M, Hirakawa M: **From genomics to chemical genomics: new developments in KEGG.** *Nucleic Acids Res* 2006, **34:**D354-357.

24. Lavin JL, Kiil K, Resano O, Ussery DW, Oguiza JA: **Comparative genomic analysis of two-component regulatory proteins in *Pseudomonas syringae*.** *BMC Genomics* 2007, **8:**397.

25. Cock PJ, Whitworth DE: **Evolution of prokaryotic two-component system signaling pathways: gene fusions and fissions.** *Mol Biol Evol* 2007, **24:**2355-2357.

26. Finn RD, Tate J, Mistry J, Coggill PC, Sammut SJ, Hotz HR, Ceric G, Forslund K, Eddy SR, Sonnhammer EL, Bateman A: **The Pfam protein families database.** *Nucleic Acids Res* 2008, **36:**D281-288.

27. Markowitz VM, Szeto E, Palaniappan K, Grechkin Y, Chu K, Chen IM, Dubchak I, Anderson I, Lykidis A, Mavromatis K, et al: **The integrated microbial genomes (IMG) system in 2007: data content and analysis tool extensions.** *Nucleic Acids Res* 2008, **36:**D528-533.
28. Galperin MY: **Structural classification of bacterial response regulators: diversity of output domains and domain combinations.** *J Bacteriol* 2006, **188:**4169-4182.
29. Wilson D, Charoensawan V, Kummerfeld SK, Teichmann SA: **DBD--taxonomically broad transcription factor predictions: new content and functionality.** *Nucleic Acids Res* 2008, **36:**D88-92.
30. Gelfand Y, Rodriguez A, Benson G: **TRDB--the Tandem Repeats Database.** *Nucleic Acids Res* 2007, **35:**D80-87.
31. Krzywinski M, Schein J, Birol I, Connors J, Gascoyne R, Horsman D, Jones SJ, Marra MA: **Circos: an information aesthetic for comparative genomics.** *Genome Res* 2009, **19:**1639-1645.
